# Biomimetic Micro-Nanostructured Evaporator with Dual-Transition-Metal MXene for Efficient Solar Steam Generation and Multifunctional Salt Harvesting

**DOI:** 10.1007/s40820-024-01612-0

**Published:** 2025-01-06

**Authors:** Ruiqi Xu, Hongzhi Cui, Na Wei, Yang Yu, Lin Dai, Xiaohua Chen

**Affiliations:** 1 College of Materials Science and Engineering, Ocean University of China, Qingdao, 266100 People’s Republic of China; 2 College of Materials Science and Engineering, Shandong University of Science and Technology, Qingdao, 266590 People’s Republic of China

**Keywords:** Double‐transition‐metal MXene, Micro-nanostructures, Solar steam generation, Salt harvesting, Multifunctionality

## Abstract

Solar-driven interfacial evaporation is one of the most attractive approaches to addressing the global freshwater shortage. However, achieving an integrated high evaporation rate, salt harvesting, and multifunctionality in evaporator is still a crucial challenge. Here, a novel composite membrane with biomimetic micro-nanostructured superhydrophobic surface is designed via ultrafast laser etching technology. Attractively, the double‐transition‐metal (V_1/2_Mo_1/2_)_2_CT_x_ MXene nanomaterials as a photothermal layer, exhibiting the enhanced photothermal conversion performance due to elevated joint densities of states, which enables high populations of photoexcited carrier relaxation and heat release, provides a new insight into the photothermal conversion mechanism for multiple principal element MXene. Hence, the (V_1/2_Mo_1/2_)_2_CT_x_ MXene-200 composite membrane can achieve a high evaporation rate of 2.23 kg m^−2^ h^−1^ under one sun, owing to the enhanced “light trap” effect, photothermal conversion, and high-throughput water transfer. Synergetically, the membrane can induce the directed precipitation of salt at the membrane edge, thus enabling salt harvesting for recycling and zero-emission of brine water. Moreover, the composite membrane is endowed with excellent multifunctionality of anti‐/de‐icing, anti-fouling, and antibacterial, overcoming the disadvantage that versatility is difficult to be compatible. Therefore, the evaporator and the promising strategy hold great potential for the practical application of solar evaporation.

**Supplementary Information:**

The online version contains supplementary material available at 10.1007/s40820-024-01612-0.

## Highlights

- The design of composite membrane with biomimetic micro-nanostructured superhydrophobic surface and (V_1/2_Mo_1/2_)_2_C MXene photothermal nanomaterials.
- The double‐transition‐metal (V_1/2_Mo_1/2_)_2_CT_x_ MXene exhibits enhanced photothermal conversion performance via the elevated joint densities of states.
- The (V_1/2_Mo_1/2_)_2_CT_x_ MXene-200 composite membrane achieves an evaporation rate of 2.23 kg m^−2^ h^−1^ under one sun, directed salt harvesting, and excellent multifunctionality of anti-/de-icing, anti-fouling, and antibacterial.

**Supplementary Information:**

The online version contains supplementary material available at 10.1007/s40820-024-01612-0.

## Graphical abstract

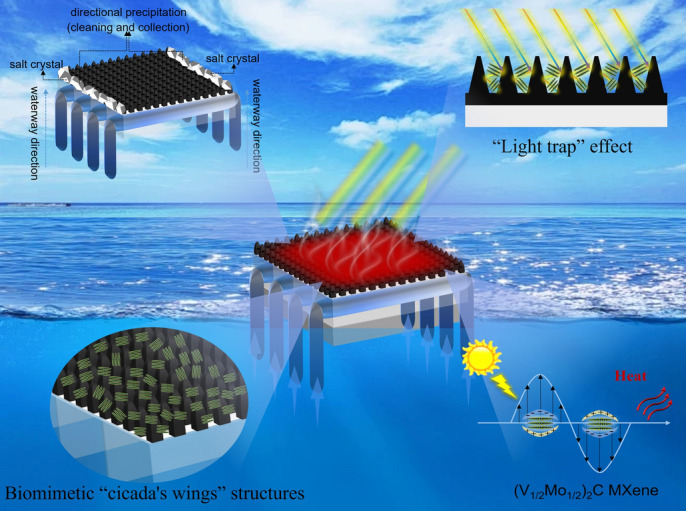

## Introduction

Freshwater is an important guarantee for the survival of all living things. However, with the overconsumption and pollution of freshwater resources, the shortage of freshwater resources has become a great challenge to the sustainable development of human society [1, 2]. Meanwhile, fossil energy pollution is becoming increasingly serious and causing grievous jeopardizing security for the environment. Therefore, solving the freshwater resources crisis and carrying out energy transformation is becoming urgent. Conspicuously, extraction of freshwater from the vast oceans utilizing desalination has become one of the most promising ways to obtain freshwater in recent years [3, 4]. However, traditional methods such as reverse osmosis and membrane distillation usually require complex infrastructure, high cost, and fossil energy consumption, which makes it difficult to meet the low-cost, portable water needs of economically underdeveloped western regions, remote island areas, ships, and households [5]. Moreover, these approaches have come under huge energy pressure in the development goals of “Carbon peaked, Carbon neutral” and will be difficult to expand in the future [6]. Therefore, a green, energy-saving, efficient, and environmentally friendly desalination method is urgently needed [7, 8].

Solar-driven interfacial evaporation technology has become one of the most attractive and sustainable solutions to the challenge of global freshwater shortage and energy crisis, due to the high energy efficiency and low energy consumption [9, 10]. However, achieving integrated high evaporation rate, salt harvesting, and multifunctionality in the practical application is still a crucial challenge. Solar evaporator consists mainly of a photothermal conversion layer, an insulating layer for thermal management, and a high-throughput water transfer channel [11–13]. Essentially, the photothermal materials, as a key component of the evaporator, determine the efficiency, lifetime, and quality of the collected freshwater. Currently, the development of photothermal materials is divided into metallic [14–16], semiconductor materials [17, 18], carbon materials [19–21], organic polymers, and some two-dimensional materials. They correspond to the main three photothermal conversion mechanisms of localized plasma surface resonance (LSPR), non-radiative relaxation, and molecular thermal vibration effects, respectively [22, 23]. Among them, MXene as a burgeoning two-dimensional material has become an excellent candidate material for desalination, due to its excellent photothermal conversion performance, abundant surface functional groups, corrosion resistance, and antimicrobial properties [24–26]. The tunability of composition, bandgap, and power function of MXene materials provide a strong guarantee for the achieving of multifunctionality in tanglesome marine environments [27, 28]. However, the further application of MXene materials in the field of solar evaporation is still limited by the poor solar absorption and oxidative failure behavior [29–31]. Thus, some previous efforts have been developed to effectively improve solar absorption over the full spectrum and improve stability, including the construction of MXene/semiconductor heterostructures [32, 33], and the decorating of carbon layer to the surface of MXene materials [34, 35]. However, these methods still have some drawbacks, such as the complexity of preparation, high volatility of composite properties, and increased costs. To solve the issues, a strategy to synthesize multiple principal elements MAX precursor and derived MXene has drawn much attention [36, 37]. In general, the properties such as optical, electrical, and magnetic of MXene materials are determined by the metal elements of the “M” site [26]. Thus, doping metal elements at the “M” site of MXene is a promising strategy to improve the electromagnetic absorption and tune the energy band structure [38]. V_2_C MXene has been confirmed to have excellent photothermal conversion, great sodiophilic, and antimicrobial performances, and can be used as an outstanding desalination candidate [39–42]. Moreover, the V atom in the “M” position of V_2_AlC is more compatible with the other elements in terms of atomic radius and electronegativity, which is very favorable to be used as the mother phase for the subsequent solid solution MAX phase material. Meanwhile, the Mo and V elements have been proven the advantages to absorb the sunlight of the ultraviolet–visible, and visible regions, respectively. Therefore, compared to the single metal element MXene, the double metal MXene of V and Mo co-doped promises to improve solar absorption and photothermal conversion performance. Although relevant MXene materials such as Mo_2.7_V_1.3_C_3_ and Mo_4_VC_4_ MXene have been reported, they are mainly used in the field of energy storage, and the optical properties and further photothermal conversion mechanisms are still unclear [43–45]. Hence, in this work, the method of doping the Mo element to the V_*n*+*1*_AlC_*n*_ MAX was employed, to attain the (V_1/2_Mo_1/2_)_*n*+*1*_AlC_*n*_ (n = 1, 2, 3) MAX phase and derived (V_1/2_Mo_1/2_)_*n*+*1*_C_*n*_T_x_ MXene. The first-principle density-functional theory (DFT) calculation method was adopted to investigate the photothermal conversion mechanisms. Moreover, to avoid oxidative failure caused by the increased high energy state in lattice distortion, thereby enhancing the stability of bimetal MXene materials, the low surface energy modification strategy of utilizing silanization instead of oxygen groups was further applied.

Noteworthily, the damage to the service life of the photothermal conversion materials from the harsh environments cannot be ignored. In the practical application of solar interfacial evaporation technology, high solar irradiation intensity, weathering, low-temperature icing, dust, and biological pollution will inevitably result in a vast reduction in the efficiency and stability of the evaporator. However, to increase the evaporation rate, most of the reported works tend to construct hydrophilic or even superhydrophilic composite materials, thereby guaranteeing an adequate water supply for the evaporation process. Unfortunately, in the continuous long-term service of the evaporator, the salts accumulation will also inevitably be generated on the hydrophilic photothermal conversion layer, leading to the decrease in the light absorption area, blockage of vapor escape channels, deterioration of evaporation performance, and the complexity of salt clean-up [46–49]. Moreover, the hydrophilic evaporator surfaces are not resistant to low-temperature icing, dust, and biological pollution accompanied by seawater. Thus, it is difficult to achieve multifunctionality in terms of salt resistance, directional salt harvesting, self-cleaning, and antimicrobial properties of the evaporator while maintaining a high evaporation rate. In nature, cicada wings have excellent superhydrophobic and self-cleaning properties due to their unique micro-nanostructure for preventing pollutants. In addition, the micro-nanostructures can also achieve the antimicrobial ability by puncturing cells [50]. Therefore, inspired by cicada wing structures, we proposed a strategy for constructing a micro-nanostructured superhydrophobic surface to collaborative achieve salt-resistant, anti‐/de‐icing, anti-fouling, and antimicrobial performances. Furthermore, the stability of porous support materials remains another key issue of concern in this area. In contrast to the insurmountable problems of photoaging and hydrolysis problems for some organic materials, ceramic materials have attracted much attention due to excellent structural and chemical stability [51, 52]. Al_2_O_3_ porous ceramic membrane has been used as a supporting layer for photothermal materials, due to its high moisture-thermal stability, remarkable corrosion resistance, chemical stability, and low price [53, 54]. Moreover, the alumina flat ceramic membrane can provide high-throughput water channels through the ample interstitial pores and parallel arrayed millimeter channels, and can be chosen as the excellent matrix material for composite membranes. Therefore, designing and constructing a composite material with excellent photothermal conversion, superhydrophobic, and stability can not only achieve efficient evaporation efficiency, but also meet multifunctionality requirements in complex and harsh marine environments, which is a great help to make solar desalination technology move toward real application.

**Fig. 1:**
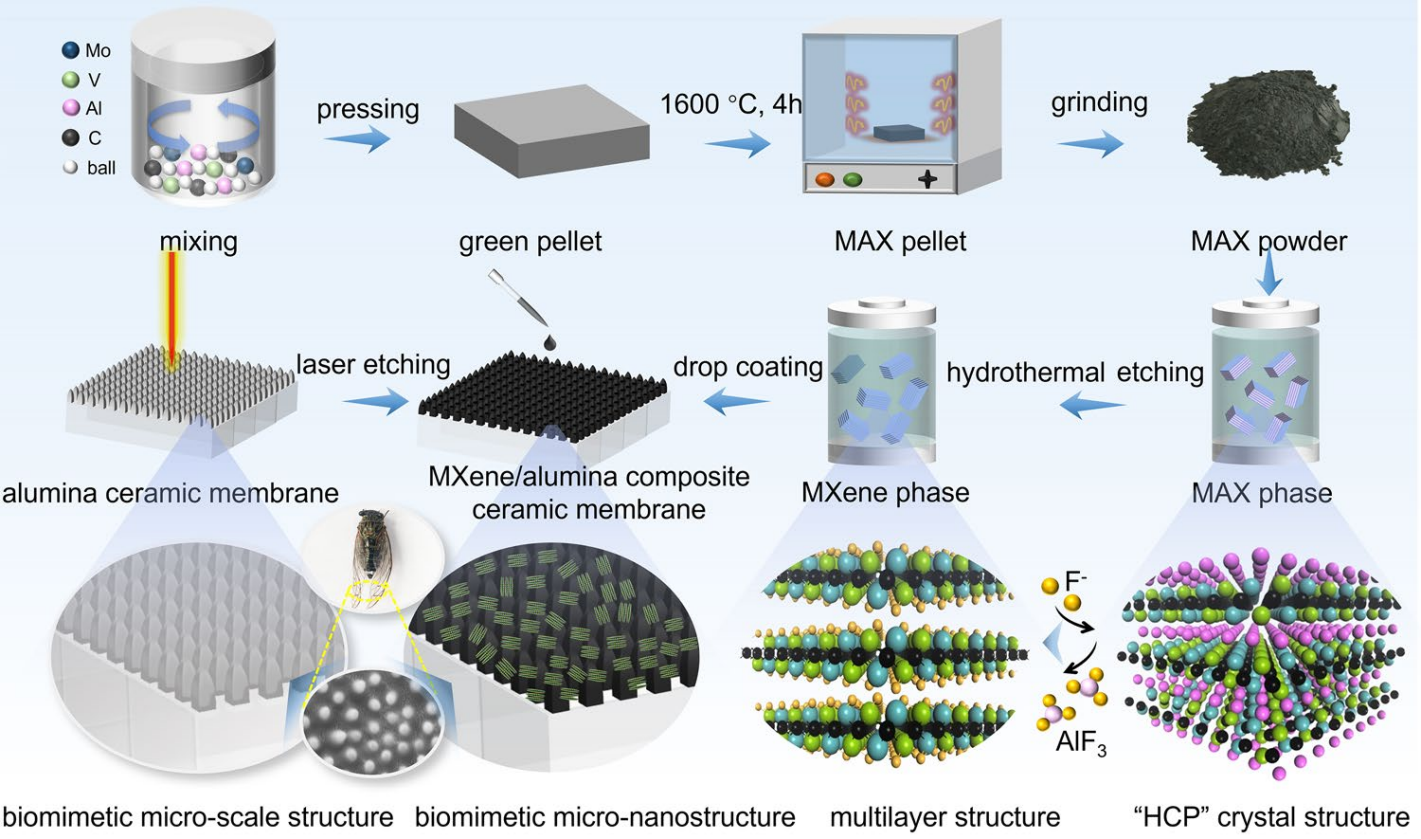
Schematic illustration of the fabrication process of (V_1/2_Mo_1/2_)_2_C MXene composite membrane with biomimetic cicada’s wings-like micro-nanostructure

In this work, inspired by cicada wing structures, a novel composite ceramic membrane with a biomimetic micro-nanostructured photothermal superhydrophobic surface was designed and constructed. The double transition metal (V_1/2_Mo_1/2_)_2_CT_x_ MXene as a photothermal conversion layer and the micro-nanostructures were fabricated via a controlled, extensible, and highly efficient ultrafast laser etching strategy, and the schematic illustration of the fabrication process is shown in Fig. 1. Thanks to the elevated joint densities of states of MXene from the doping of the Mo element, the photothermal conversion performance is significantly enhanced by the high populations of hot electrons and holes to be produced through photoexcitation. Moreover, the micro-nanostructure is not only capable of exerting a “light-trapping” effect to promote solar absorption, but also of utilizing a “mechanical interlocking” effect to improve the bonding strength between the ceramic matrix and the MXene nanomaterials. More importantly, the (V_1/2_Mo_1/2_)_2_CT_x_ MXene-200 composite ceramic membrane can achieve a high evaporation rate of 2.23 kg m^−2^ h^−1^ under one sun irradiation, owing to the enhanced solar absorption, excellent photothermal conversion, as well as the high-throughput water transfer channels of ceramic membrane. In particular, the superhydrophobic surface prevents the seawater film formation on the membrane surface, and promotes the directional salt precipitation at the membrane edge, resulting in salt harvesting and zero-emission of highly concentrated brine water. Furthermore, benefiting from the reduced contact area between the water, pollutants, and the micro-nanostructured surface, the composite ceramic membrane can achieve anti‐/de‐icing, anti-fouling, and antibacterial, breaking the disadvantage that versatility is difficult to be compatible.

## Experimental Section

### Materials

Lithium fluoride (LiF, AR, 99%) and perfluorooctyltrimethoxysilane (POTS, AR, > 97%) were purchased from Shanghai Aladdin Biochemical Technology Co. Ltd. Ethanol (C_2_H_6_O, AR, > 99.7%) and ethyl acetate (C_4_H_8_O_2_, AR, 99.5%) were bought from Sinopharm Chemical Reagent Co. Ltd. Polydimethylsiloxane (PDMS, SYLGARD 184) and matching curing were acquired from DOW CORNING Co. Ltd. Deionized water used in all experiments was purified by V6-201A-A water purification system (Hong Sen Environmental Technology Co. Ltd.).

### Preparation of MAX, MXene, and Micro-Nanostructured Composite Membranes

#### ***Preparation of (V***_***1/2***_***Mo***_***1/2***_***)***_***n***+***1***_***AlC***_***n***_*** MAX***

The (V_1/2_Mo_1/2_)_*n*+*1*_AlC_*n*_ (n = 1, 2, 3) MAX was synthesized by the following procedure. Molybdenum (99.9%, Leber Metal Materials Ltd., -300 mesh), vanadium (99.9%, Leber Metal Materials Ltd., -300 mesh), aluminum (99.5%, Leber Metal Materials Ltd., -300 mesh), and graphite (99.95%, Sinopharm Chemical reagent Co. Ltd., -600 mesh) powders were weighted with the molar ratio of V: Mo: Al: C = 1:1:1:1, V: Mo: Al: C = 1.5:1.5:1:2, V: Mo: Al: C = 2:2:1:3, respectively, and then mixed in a ball mill jar and ball milled for 20 h at 100 rpm with a 2:1 ball: powder mass ratio. Then, the mixed powders were put into a mold with d = 20 mm and pressed into dense green cubes under a pressure of 40 MPa. Subsequently, the obtained green cubes were heated to 1600 °C with a heating rate was 3 °C min^−1^ in an alumina crucible with 800 cm^3^ min^−1^ flowing argon in a vacuum high-temperature furnace and held for 4 and 5 h, respectively, and then passive cooling to room temperature. After taking out the sample, the sintered blocks were crushed with a cemented carbide hammer and then ground to 300 mesh particles in an agate mortar with a grinding pestle.

#### ***Preparation of Derived (V***_***1/2***_***Mo***_***1/2***_***)***_***n***+***1***_***C***_***n***_*** MXene***

The MXene derived from the (V_1/2_Mo_1/2_)_*n*+*1*_AlC_*n*_ (n = 1, 2, 3) MAX was synthesized by hydrothermal-assisted in situ hydrofluoric acid etching. Specifically, the etching solution consisted of hydrochloric acid (40 mL, 12 M), deionized water (20 mL), and LiF (3 g). Then, the MAX powders (2 g) were weighed and slowly put into the etching solution in a polytetrafluoroethylene reactor. Subsequently, the sealed reactor was held in an oven at 100 and 120 °C for 5 days, respectively, and then cooled to room temperature. After the reaction, decanting the acidic supernatant, the sediment was redispersed in deionized (DI) water to be washed out by repeating centrifugations at 4500 rpm, until the pH = 6 of the supernatants. Finally, the black precipitate was held in a vacuum oven at 40 °C for 24 h to collect, obtaining the multilayered MXene.

#### Preparation of Biomimetic Micro-Nanostructured Composite Membranes

Al_2_O_3_ flat ceramic membrane with a size of 30 × 30 mm^2^ prepared by our group was used in this work. The frequency of the pulsed laser is set to 20 MHz, the pulse width is 5000 ns, the scanning speed is 100 mm s^−1^, the laser power is 70%, the laser spot diameter is 1 mm, and the line spacing is 100 ~ 400 μm. In the laser etching process, the laser light source carried out the microstructures processing on the Al_2_O_3_ flat ceramic membrane surface. The micro-convex column structure was attained and controlled by adjusting the line spacing of the pattern. To enhance the stability and dispersion of MXene in PDMS solution, preliminary modification of MXene was carried out using POTS. Specifically, the multilayer (V_1/2_Mo_1/2_)_2_C MXene powders were weighed 50, 100, and 200 mg, mixed with perfluorooctyltrimethoxysilane (POTS) (400 mg) and ethanol (35 mg), respectively. Then, the mixed solutions were magnetic stringed at room temperature for 1 h. Subsequently, after centrifugation of the homogeneous solution at 9000 rpm for 10 min, the lower precipitate was taken to obtain the hydrophobically modified multilayer (V_1/2_Mo_1/2_)_2_C MXene nanopowders. Then, the diluted PDMS solutions were obtained by the following procedures: Polydimethylsiloxane (PDMS) (150 mg) and matching curing (10 mg) were weighed and added to ethyl acetate (10 g), and magnetically stirred until the PDMS solution was dispersed. Next, the hydrophobically modified multilayer (V_1/2_Mo_1/2_)_2_C MXene nanopowders were added to the above diluted PDMS solution (5 mL), and continued magnetically stirred for 2 h to obtain a viscous well-dispersed homogeneous PDMS/(V_1/2_Mo_1/2_)_2_C MXene homogeneous solution. Finally, the solution obtained above was drop-coated onto the surface of the alumina ceramic membrane with 100 μm spacing of micro-convex column structure. And the ceramic membranes were placed in an oven at 120 °C for 3 h to cure the PDMS, thereby achieving the biomimetic micro-nanostructured composite membranes of (V_1/2_Mo_1/2_)_2_C MXene-50, (V_1/2_Mo_1/2_)_2_C MXene-100, and (V_1/2_Mo_1/2_)_2_C MXene-200.

### Characterization

An X-ray diffractometer (XRD, Bruker D8 ADVANCE, scan speed was 4° min ^−1^) was used to characterize the phase composition of the samples by using a Cu Kα radiation source. The micromorphology of the samples was carried out on field-emission scanning electron microscopy (ZEISS-Gemini SEM300). The attached EDS spectrometer was used to analyze the element distribution. A high-resolution transmission electron microscope (JEOL TEM 2100F) was used to study the crystal structure of the (V_1/2_Mo_1/2_)_2_CT_x_ MXene. The surface chemical status of the elements was characterized by X-ray photoelectron spectroscopy (XPS, Thermo Scientific K-Alpha). The wetting performance of the membrane was carried out on a Contact Angle Meter (JC2000D). A spectrometer (3600plus, Shimadzu, Japan) was used to measure the UV–Vis-NIR diffuse reflectance spectra using BaSO_4_ as the reflectance standard. Fourier transform infrared spectroscopy (Thermo Scientific Nicolet iS20) has been used to find out the chemical structures of samples, the spectrum ranges from 4000 to 500 cm^−1^. Laser confocal microscopy (KEYENCE, VK-X250, Japan) was used to observe the three-dimensional morphology of membrane surfaces. A high-speed camera (Acuteye, V4.0) was used to record snapshots of the water droplet during fall and rebound.

## Results and Discussion

### Preparation and Characterization

**Fig. 2:**
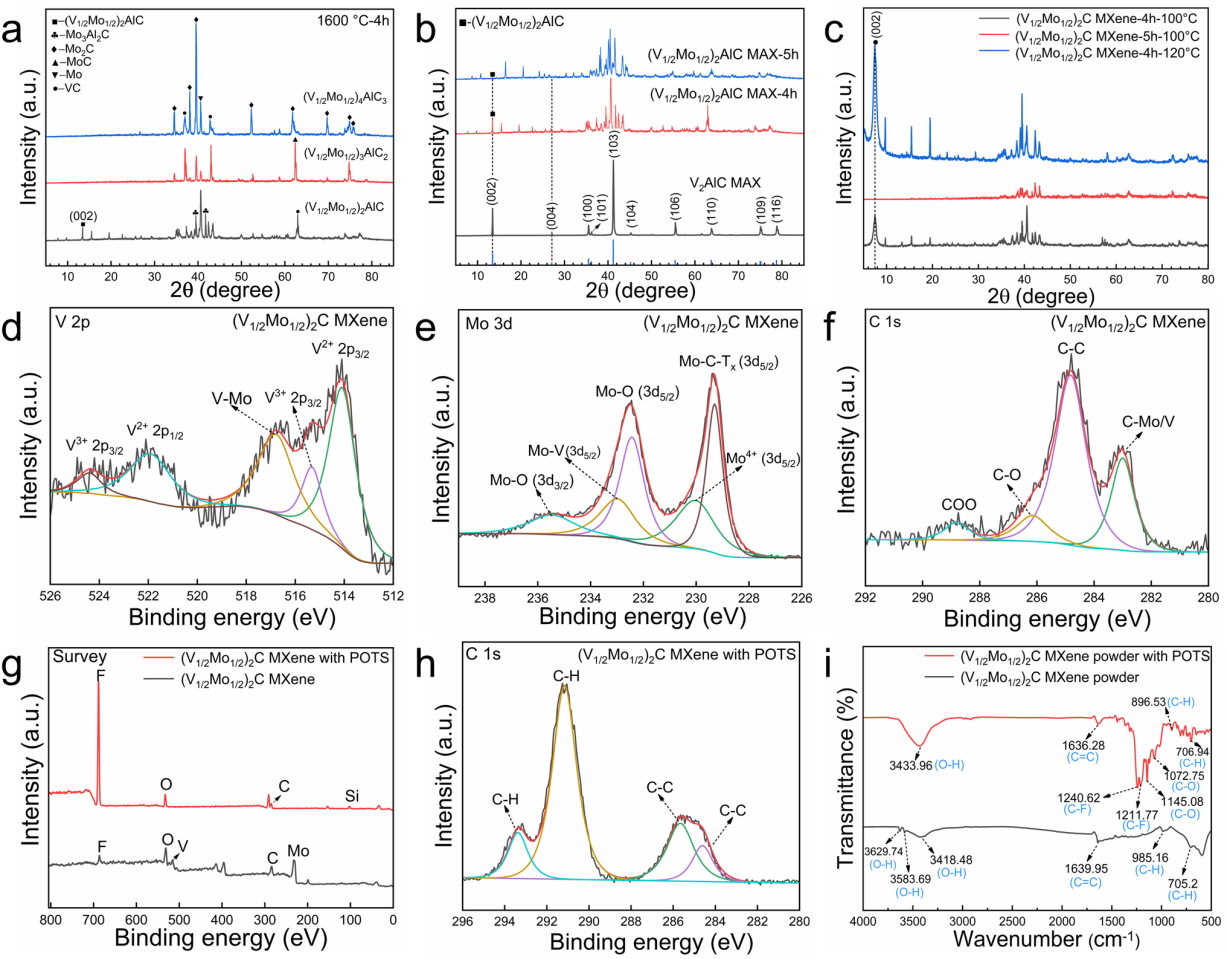
Characterization of MAX phase and derived multilayer MXene: **a** XRD patterns of MAX phase with different configurations at 1600 °C for 4 h, **b** MAX phase of (V_1/2_Mo_1/2_)_2_AlC under different sintering time, **c** (V_1/2_Mo_1/2_)_2_CT_x_ MXene under different etching conditions. XPS and FT-IR characterization of (V_1/2_Mo_1/2_)_2_CT_x_ MXene before and after POTS modification: **d-f** spectrums of V 2*p*, Mo 3*d*, and C 1*s* before modification, **g** survey spectrum of (V_1/2_Mo_1/2_)_2_CT_x_ MXene before and after modification. **h** spectrum of C 1*s* after modification, **i** FT-IR transmittance spectrum of (V_1/2_Mo_1/2_)_2_CT_x_ MXene before and after modification

To analyze the effects of different configurations and sintering time on the MAX phase formation, firstly, the phase compositions of MAX with different configurations were investigated at a sintered temperature of 1600 °C for 4 h. As shown in Fig. 2a, at the same sintering temperature and time, the MAX phase emerged in the “211” configuration sample, and the “312” and “413” samples did not show the typical MAX diffraction peaks and contained ample impurities, including Mo_2_C, MoC, VC, and Mo_3_Al_2_C. Meanwhile, the content of impurity phases, especially molybdenum-containing carbides, increased significantly with the increase in the ratio of metal and C elements. In addition, the effect of sintering time on the MAX phase formation was further investigated. At the same temperature, when the sintering time was extended to 5 h, the impurity phases were significantly higher in all three samples with different ratios, and the typical MAX diffraction peaks appeared only for the “211” ratio as well (Fig. S1). The results showed that only the “211” configuration was capable of generating the MAX phase for the Mo, V system with iso-atomic ratio, whereas as the content of the metal and C element increased, the carbide ceramic phase was easier to generate, which is thermodynamically competitive caused by the smaller electronegativity of metal and C elements. The comparison of the V_2_AlC parent phase and the (V_1/2_Mo_1/2_)_2_AlC MAX phase at different sintering time in the same “211” configuration is shown in Fig. 2b. The (002) peak occurred at 13.47° in V_2_AlC MAX, while in (V_1/2_Mo_1/2_)_2_AlC MAX-4 h, it was slightly shifted toward a lower angle to the 13.43°, indicating an increase in the interplanar spacing of the double metals MAX phase, further proving the formation of the solid solution containing Mo element. For the (V_1/2_Mo_1/2_)_2_AlC MAX-5 h, there was only a tiny (002) peak appeared. Further, the (V_1/2_Mo_1/2_)_2_AlC MAX-4 h and (V_1/2_Mo_1/2_)_2_AlC MAX-5 h were etched by hydrothermal-assisted in situ hydrofluoric acid etching method to attain derived MXene. As shown in Fig. 2c, under the same etching conditions (hydrothermal temperature was 100 °C), the (V_1/2_Mo_1/2_)_2_AlC MAX-4 h sample showed the typical (002) diffraction peak of MXene at 7.47°. But almost no diffraction peaks of MXene can be detected in the (V_1/2_Mo_1/2_)_2_AlC MAX-5 h sample, which may be attributed to the strong metal bonding between Mo, V, and Al atomic plane that leads to difficult etching of the Al layer. To improve the MXene purity, the hydrothermal temperature was further increased to 120 °C to remove the impurity phase. The results showed that the (002) diffraction peak intensity was significantly larger than that of the impurity phase, which means that the obtained MXene material can be further utilized. Therefore, in the subsequent characterization tests and preparation of the composite ceramic membranes, the MXene materials used were all the derived (V_1/2_Mo_1/2_)_2_CT_x_ MXene-4 h at an etching temperature of 120 °C, abbreviated as (V_1/2_Mo_1/2_)_2_CT_x_ MXene.

X-ray photoelectron spectroscopy was used to analyze the chemical states of (V_1/2_Mo_1/2_)_2_CT_x_ MXene. The V 2*p* region can be fitted with five peaks at 514.08, 515.31, 516.78, 521.9, and 524.4 eV, corresponding to V^2+^, V^3+^, V-Mo, V^2+^, and V^3+^, respectively, as shown in Fig. 2d. Most peaks in the V 2*p* region are assigned to V bonded to C atoms in the MXene. The oxidation states of V might come from the different surface terminations of O_2_ or OH^−^ bonded on the V atom on the surface. Thus, the above results indicated that V was located in different transition metal layers to bond to C and other terminations, further implying the formation of a double-metal solid solution [43]. Similarly, the Mo 3*d* region (Fig. 2e) can be fitted to five peaks, the peak at 229.3 eV corresponds to Mo-C-T_x_, the peaks at 230.03, 232.43, and 235.46 eV might come from the oxidation states of Mo in the Mo^4+^, which corresponds to the little amount of oxides presented, and the peak at 232.97 eV belonged to the Mo-V, further indicating the solid solution of Mo element. The C 1*s* region (Fig. 2f) can be fitted to four peaks, corresponding to C-Mo/V, C–C, C-O, and C-OO at 283.01, 284.82, 286.15, and 288.9 eV, respectively. To further improve the stability of MXene in the seawater environment and dispersibility in PDMS solution, according to the similarity-intermiscibility theory, the modification strategy of silanization was performed on the (V_1/2_Mo_1/2_)_2_C MXene by POTS. As shown in Fig. 2g, the elements of V and Mo on the modified MXene surface were unable to be observed, the new signal of the Si element appeared, and the peak intensity of the F element was significantly increased. Further, the XPS spectra of V, Mo, and C elements in modified MXene were further analyzed, whereas the characteristic peaks of V and Mo did not appear (Fig. S2). This result can be attributed to the peaks of the V and Mo elements being covered by the C, F, O, and Si elements from the POTS, so far as to be undetectable by the XPS instrument. The C 1*s* region can be fitted to four peaks at 284.6, 285.6, 291.1, and 293.3 eV, corresponding to C–C, C–C, C-H, and C-H bonds, respectively (Fig. 2h). Moreover, the surface terminal groups of MXene powder before and after POTS modification were further studied by FT-IR spectroscopy. As shown in Fig. 2i, the peaks of O–H, C = C, and C-H were detected in MXene before modification, which might come from the -OH groups, whereas the new peaks of C-F and C-O arose in the modified MXene, belonging to the POTS. Therefore, the XPS and FT-IR results demonstrated the successful grafting of (V_1/2_Mo_1/2_)_2_C MXene by POTS.

**Fig. 3:**
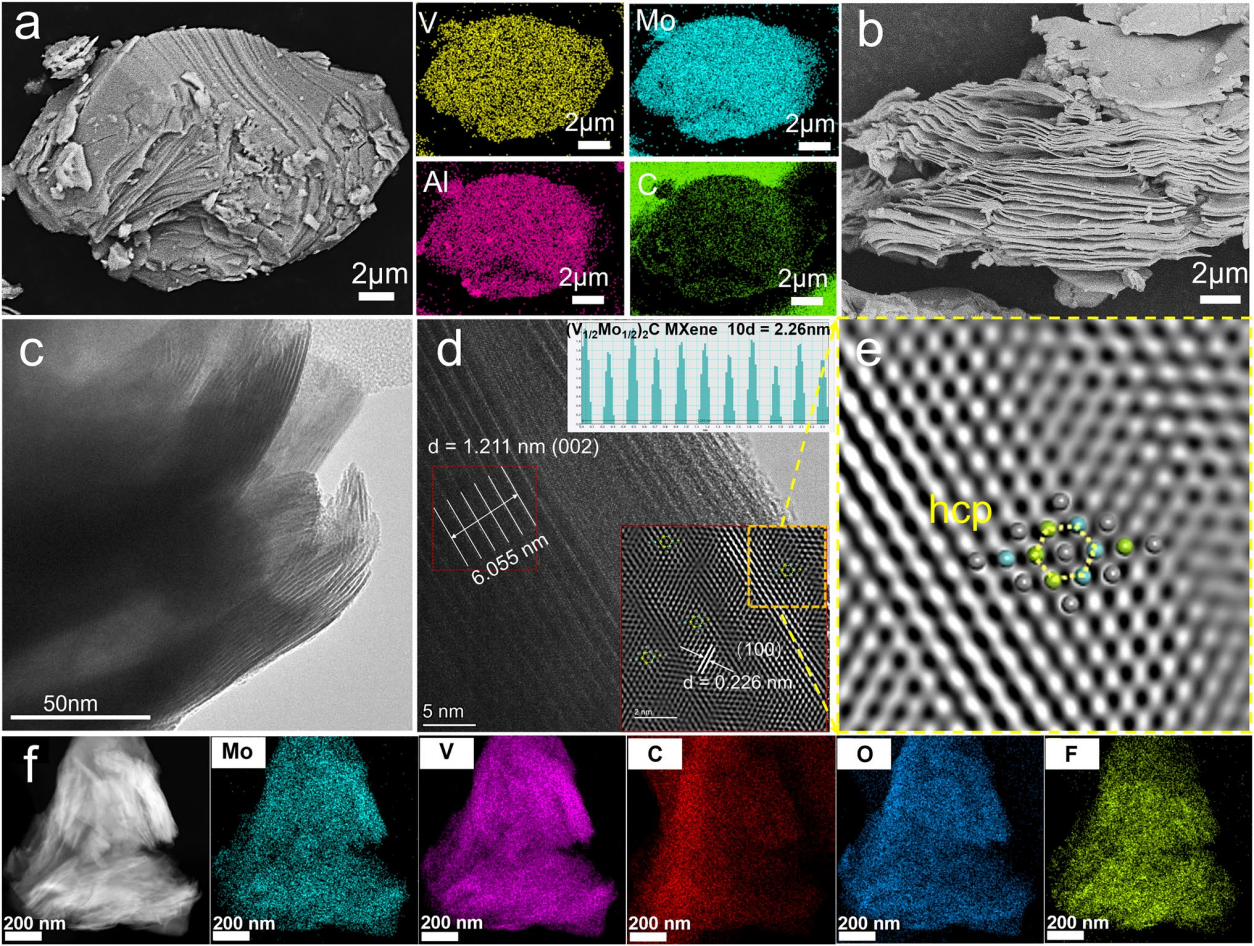
**a** SEM and EDS images of (V_1/2_Mo_1/2_)_2_AlC MAX phase under sintering temperature at 1600 °C for 4 h. **b** Derived (V_1/2_Mo_1/2_)_2_CT_x_ MXene-4 h at etching temperature of 120 °C. **c-f** HRTEM and EDS images of **b**: **c** multilayer morphology of MXene, **d** lattice fringes and interplanar spacing, **e** “hcp” crystal structure corresponds to the (002) crystal plane of MXene, **f** EDS images of (V_1/2_Mo_1/2_)_2_CT_x_ MXene

Further, the microscopic morphology of the MAX phase under different ratios and sintering conditions was observed by SEM to analyze the formation behavior. At the sintered temperature of 1600 °C for 4 h, the MAX phase of the “211” and “312” configurations, exhibited a typical layered structure, whereas the “413” structure showed an integral block (Figs. 3a and S3a-b). In addition, the EDS results of (V_1/2_Mo_1/2_)_2_AlC MAX-4 h showed the uniform distribution of Mo, V, Al, and C elements. Noteworthily, when the sintering time was extended to 5 h under the same temperature, the typical layered structure appeared only for the “211” ratio, and a new porous microscopic morphology emerged in the other configurations (Fig. S4a-c). As the proportion of metal and C elements increases, the particles with a porous structure appear at first, and larger-sized grains tightly bound are formed. The results may be attributed to the “Kirkendall holes” created by atomic diffusion in the “312” sample, and lots of carbides close to each other grow in the “413” sample. Therefore, the (V_1/2_Mo_1/2_)_2_AlC MAX can be achieved from samples of “211”-4 h and “211”-5 h, consistent with the XRD results. The microscopic morphology of derived MXenes from the MAX under different etching conditions was further observed. All MXene exhibited a typical accordion-like multilayered structure after the etching of the Al layer (Figs. 3b and S5a-b), demonstrating the successful acquisition of derived MXene materials.

To further investigate the crystal structure of the obtained MXene material, a TEM characterize test was performed on the (V_1/2_Mo_1/2_)_2_C-4 h-120 °C sample (Fig. 3c-e). The sample exhibited a distinct multilayer stacking structure (Fig. 3c), and the interplanar spacing was calculated to be 1.211 nm (layer spacing) and 0.226 nm (Fig. 3d), corresponding to the typical (002) and (100) planes of (V_1/2_Mo_1/2_)_2_ MXene (the detailed description of the calculations process is shown in Supporting Information). Further, the crystal structure model of (V_1/2_Mo_1/2_)_2_C MXene was constructed based on first principles calculation, and crystal planes with different orientations were cut to match validation with the actual atomic surfaces. Appropriately, the result showed that the (002) crystal plane of the model can be able to highly correspond to the exposed atomic surface, and presented a representative basal plane hexagonal symmetry structure (Fig. 3e). Moreover, the EDS test was used to detect the element distribution of MXene and quantify the atomic ratio of transition metal elements. As shown in Fig. 3f, the uniform distribution of Mo, V, C, O, and F elements can be detected, which further demonstrates the successful preparation of a solid solution of (V_1/2_Mo_1/2_)_2_CT_x_ MXene and the presence of surface terminal groups such as -OH, and -F. The atomic ratio of all elements was obtained from EDS results, as shown in Table S1; the atomic ratio of V: Mo was about 1.17: 1, approaching the equimolar ratio, whereas the loss of Mo element can be attributed to the generation of ceramic phases containing molybdenum, which agrees with the XRD results. In addition, the tested amount of C element was higher than the total metal, which is due to the affection of the carbon support film. Noteworthily, the presence of oxygen and fluorine elements proved oxygen- and fluorine-containing terminal groups again, and the amount of F surface termination was very small.

### Construction and Photothermal Performance of Composite Ceramic Membranes

**Fig. 4:**
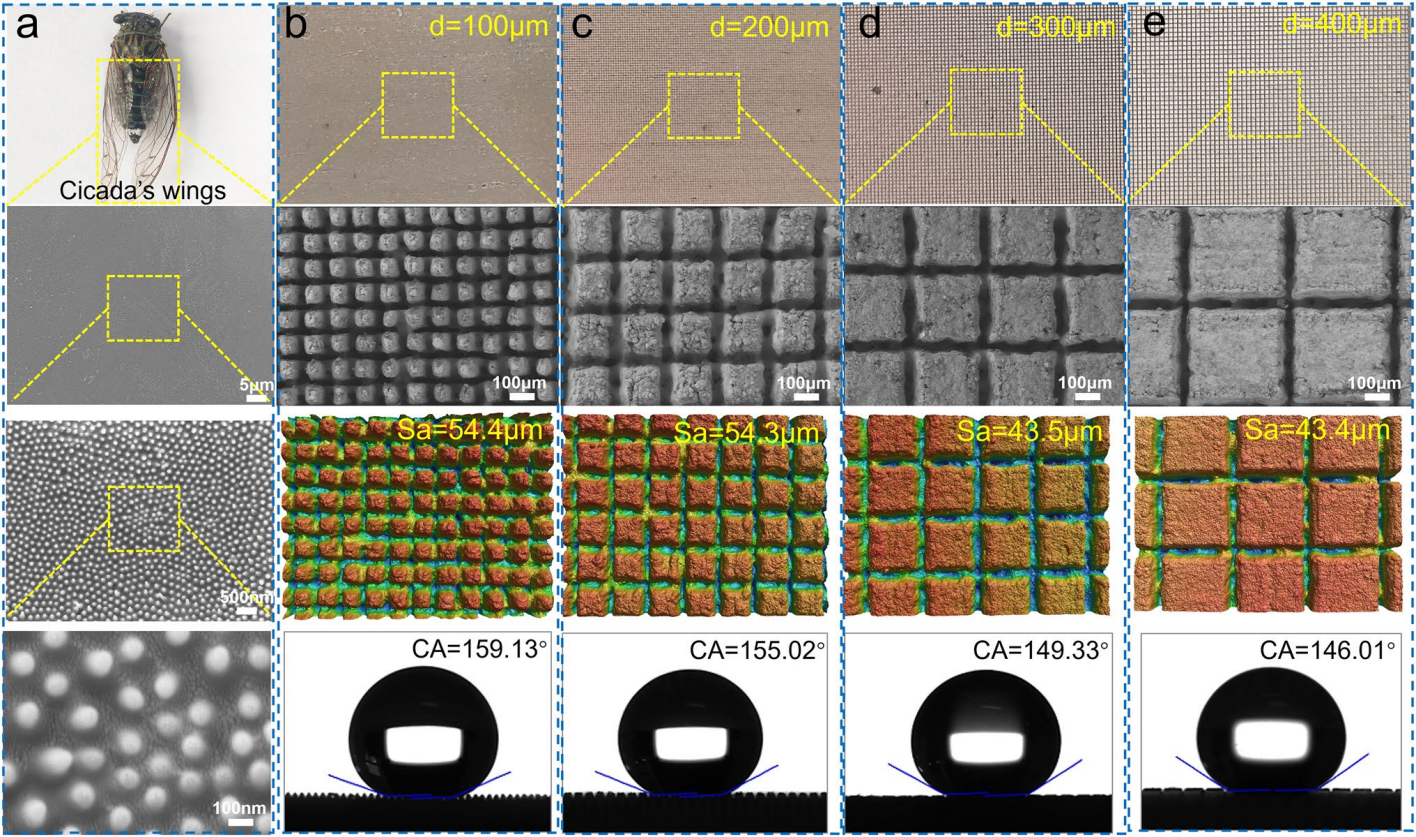
**a** Photograph and SEM images of cicada’s wings. **b-e** Photographs, SEM images, surface three-dimensional morphology, surface roughness, and contact angles of alumina ceramic membrane surfaces with different microstructure spacing of **b** d = 100 μm, **c** d = 200 μm, **d** d = 300 μm, and **e** d = 400 μm

The construction of superhydrophobic surfaces usually needs two steps: the design of the surface micro-nanostructured structure and the modification of the low surface energy. Inspired by the superhydrophobic cicada’s wings, the natural surface micro-convex structures caught our attention, as shown in Fig. 4a. To realize the fabrication of the biomimetic structures, the ultrafast laser etching strategy with high efficiency, portability, flexibility, and scalability was performed on a flat alumina ceramic membrane. Based on the surface superhydrophobic theory, the micro-nanostructure spacing significantly affects the construction of superhydrophobic models. Thus, the patterning spacing was varied from 100 to 400 μm, and the morphology, surface roughness, and wettability of different microstructured surfaces before and after PDMS modification were further investigated. All the ceramic membranes exhibited a macroscopic regular grid shape, and the microscopic morphology gradually converted from a micro-convex columnar structure to a planar “field” structure with the increase in the pattern spacing. The surface roughness of the membranes also decreased from 56.2, 55.7, 47.5, to 45.4 μm (Fig. S6), and all of them displayed superhydrophilicity (CA = 0°), corresponding to the patterning spacing of 100, 200, 300, and 400 μm, respectively. Subsequently, the low surface energy modification of different microstructured surfaces was carried out by quantitative PDMS dilution solution. As shown in Fig. 4b-e, although the surface roughness of all modified ceramic membranes decreased slightly, all of them exhibited hydrophobic properties, indicating that the silane groups can contribute to the hydrophobicity of the membranes. Moreover, the contact angles of the ceramic membrane surface increased with the increase in surface roughness, demonstrating the improvement the hydrophobicity. Noteworthily, the superhydrophobic surface was only achieved when the patterns spacing at 100 and 200 μm under the same modification conditions, corresponding to the contact angles of 159.13° and 155.02°, which can be attributed to the construction of biomimetic cicada’s wing-like micro-convex structures. Therefore, to obtain the best superhydrophobicity, the modified ceramic membrane with pattern spacing of 100 μm was used as a substrate for further study.

**Fig. 5:**
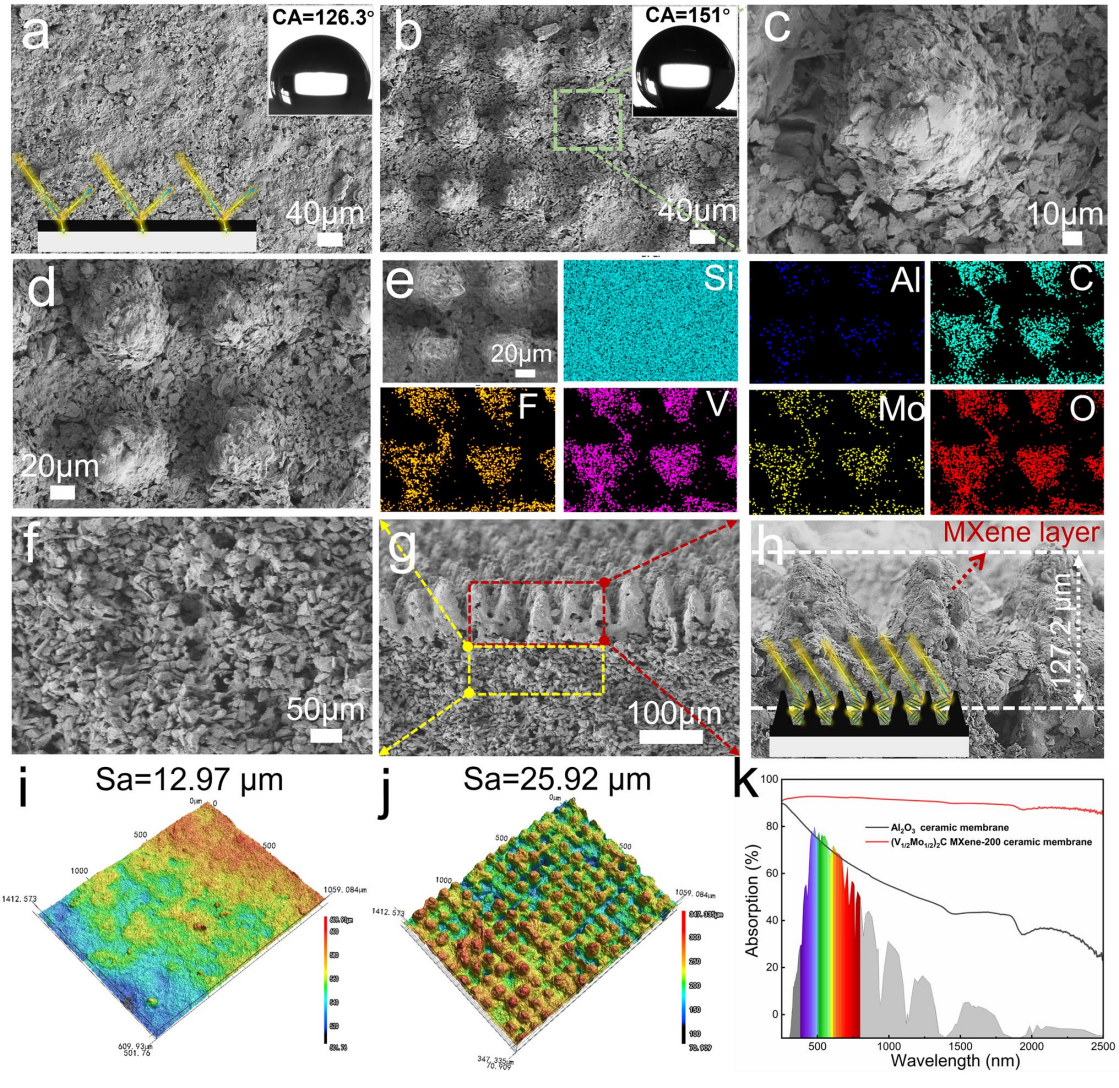
SEM images and surface contact angle pictures of MXene/alumina composite ceramic membranes: **a** (V_1/2_Mo_1/2_)_2_C MXene-200 composite ceramic membrane without grove structure,** b** (V_1/2_Mo_1/2_)_2_C MXene-200 composite ceramic membrane with groove distance of 100 μm. **c** SEM images of MXene distribution on a single column. **d, e** A partial enlargement and EDS images of **b**. **f** Three-dimensional interconnected porous structure at the bottom of the composite ceramic membrane. **g** Overall cross-sectional morphology of composite ceramic membrane. **h** Cross-sectional morphology of micro-nano-convex columns of composite ceramic membrane, and schematic illustration of the “light trap” effect. **i, j** Surface three-dimensional morphology and surface roughness of **i** (V_1/2_Mo_1/2_)_2_C MXene-200 composite ceramic membrane without grove structure, **j** (V_1/2_Mo_1/2_)_2_C MXene-200 composite ceramic membrane with groove distance of 100 μm. **k** Absorption spectrums of alumina ceramic and (V_1/2_Mo_1/2_)_2_C MXene-200 composite ceramic membranes

To obtain a composite ceramic membrane with photothermal superhydrophobic surface, hydrophobically modified (V_1/2_Mo_1/2_)_2_C MXene/PDMS solution was drop-coated onto the surface of the modified alumina ceramic membrane described above. Meanwhile, the content of MXene was adjusted from 50 to 200 mg, to investigate the hydrophobicity and photothermal conversion property change of the membranes. As shown in Fig. 5b-e, the membrane surface exhibited a micro-convex columnar structure with upper stacked layered nano-MXene. The EDS images demonstrated the uniform distribution of Al, O, C, V, Mo, F, and Si elements, belonging to the alumina ceramic substrate, MXene, and PDMS. Under different MXene contents, all three types of composite membranes can achieve superhydrophobic with contact angles of 154.4°, 151.9°, and 151°, corresponding to MXene additions of 50, 100, and 200 mg, respectively (Figs. 5b and S7a-b). In addition, to further validate the effects of micro-nanostructures, the (V_1/2_Mo_1/2_)_2_C MXene-200 membrane without groove structure was fabricated as well, and the contact angle on its surface was only 126°, displaying a hydrophobic effect rather than a superhydrophobic effect (Fig. 5a). The difference between the contact angles can be explained by employing the Cassie–Baxter equation [55].

1 $$  \cos {\mkern 1mu} \theta _{{\text{C}}}  = f{\mkern 1mu} \cos {\mkern 1mu} \theta _{{\text{Y}}}  + f - 1 $$,

wherein *θ*_Y_ is Young’s contact angle, and ƒ is the liquid–solid contact fraction. According to the equation, the decreased contact area between the water droplet and the surface can enhance the increase in the apparent contact angle *θ*_C_. Therefore, the forming mechanism of the superhydrophobic surface can be concluded that the micro-convex columnar structure on the surface is the first micrometer-scale structure, and the overlaying multilamellar layer of MXene materials form the second nanometer-scale structure, and then the PDMS coating provides an essential condition of low surface energy, thus achieving the construction of the superhydrophobic surface. In addition, the contact angles displayed a slight decrease with the MXene content increased, which is attributed to the micron-sized pores being gradually filled, resulting in the surface roughness decreasing. However, (V_1/2_Mo_1/2_)_2_C MXene-200 composite ceramic membrane presented the best dispersion and uniformity of MXene. Thus, the overall structure of the (V_1/2_Mo_1/2_)_2_C MXene-200 composite ceramic membrane was further investigated. As shown in Fig. 5f-h, the bottom exhibited a micron-sized interstitial porous structure inherited from the original ceramic membrane, and the columnar micro-convex structures were neatly arranged in arrays on the surface with a height of 127.2 μm, and the generation of the array interstices was alumina particles ablated away by the laser, so the columnar structures were derived from the original ceramic membrane rather than being added on to it, guarantying a strong bonding of the columnar micro-convex structures to the ceramic substrate. The top structure of the composite membrane was the cladding of MXene materials (Fig. 5h). The MXene materials with lamellar structure were simultaneously distributed uniformly on surface columnar protrusions and grooves via the stickiness of PDMS, but not filling the grooves due to small size. The results can be attributed to that the groove structure can induce the flow of MXene/PDMS liquid to achieve uniform distribution, and can effectively improve the bonding strength through the “mechanical interlocking” effect of grooves and nanoparticles as well. To further verify the bonding strength of MXene/PDMS to the ceramic substrate, ultrasound and sandpaper sanding scratch experiments were further implemented on the (V_1/2_Mo_1/2_)_2_C MXene-200 composite ceramic membrane. Under 60-min continuous sonication at 40 kHZ, the MXene powders were not dropped off in the water (Fig. S7c). Meanwhile, after scratching with 800 mesh sandpaper, the MXene/PDMS coating was still not peeling off from the ceramic substrate and remained superhydrophobic (Fig. S7d). Therefore, the biomimetic columnar micro-convex-groove multistage structures can provide a great guarantee for the binding of MXene materials and the stability of superhydrophobic surfaces.

The light absorption performance of the composite ceramic membranes was further investigated. To verify the influence of surface micro-nanostructures on the optical properties of composite membranes, the solar absorbance with and without micro-nanostructure at the same MXene content was tested. Compared with the (V_1/2_Mo_1/2_)_2_C MXene-200 composite ceramic membrane without micro-nanostructure, the micro-nanostructured (V_1/2_Mo_1/2_)_2_C MXene-200 membrane exhibited higher solar absorbance (Fig. S8b). The reason can be concluded that the “light trap” effect of surface micro-nanostructure (inset of Fig. 5h) [56], which allows the refraction of incident light back and forth in the columnar micro-convex structures and the interlayer structure of MXene, increasing the light absorbance path length, thereby enhancing the solar absorption. In contrast, a sharply changing refractive index at the interface between smooth and air will create a typical Fresnel reflection, which leads to a higher reflectivity, thus resulting in the decrease in absorption (inset of Figs. 5a and S8c). Moreover, this viewpoint was further verified by the surface three-dimensional morphology and roughness with different structures. The surface roughness of membranes presented an increasing trend from 7.03, 12.97, and 25.92 μm, corresponding to the original alumina flat ceramic membrane, (V_1/2_Mo_1/2_)_2_C MXene-200 composite ceramic membrane without micro-nanostructure, and micro-nanostructured (V_1/2_Mo_1/2_)_2_C MXene-200 composite ceramic membrane (Figs. 5i-j and S8a). Eventually, the original alumina flat ceramic membrane displayed a high reflectance of 48.52% and low solar absorption of 51.4% in the full spectral range of 250–2500 nm, whereas the (V_1/2_Mo_1/2_)_2_C MXene-200 composite membrane with micro-nanostructure has only 9.89% reflectance and exhibited the absorption of 90.1% (Fig. 5k), which is attributed to the great solar absorption properties of MXene and the synergistic superposition “light trap” effect of micro-nanostructures.

**Fig. 6:**
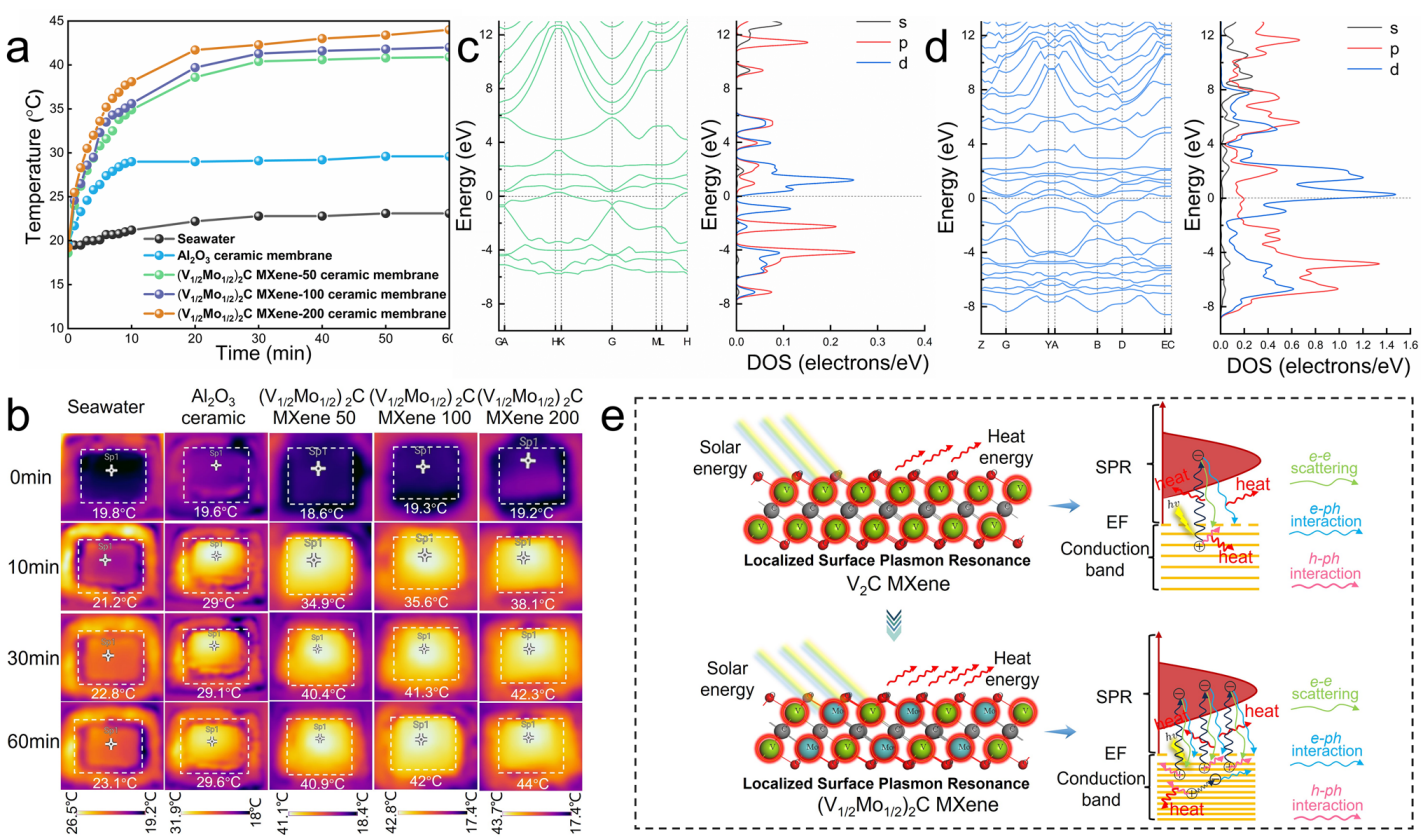
**a** Surface temperature curves of samples over time under one sun irradiation (1.0 kW m^−2^). **b** IR images of all samples. The energy band structure and electronic state density (DOS) of **c** V_2_CO_2_ MXene under 1 layer and **d** (V_1/2_Mo_1/2_)_2_CO_2_ MXene under 1 layer (I type). **e** Schematic diagram of the photothermal conversion mechanisms of the V_2_C MXene and (V_1/2_Mo_1/2_)_2_C MXene

Further, the photothermal conversion performance of the composite ceramic membranes, as the key safeguard for solar evaporation and photothermal de-icing, was investigated under one sun irradiation. Compared with pure seawater and original alumina ceramic flat membrane, the three composite membranes with MXene showed a fast photothermal response and excellent photothermal conversion performance (Fig. 6a-b). Meanwhile, the photothermal response speed and photothermal conversion temperature of the composite ceramic membranes were gradually improved with the increase in MXene content. Especially, the (V_1/2_Mo_1/2_)_2_C MXene-200 composite membrane reached a wet surface temperature of 44 °C under one sun irradiation at 60 min, indicating the excellent photothermal conversion property of (V_1/2_Mo_1/2_)_2_C MXene material. In contrast, the monometallic element V_2_C MXene only reached 36.9 °C at the same content (Fig. S9). The results further demonstrated the enhancement of MXene’s photothermal conversion property by doping with double transition metal elements.

The photothermal conversion mechanism of MXene materials can be attributed to the localized surface plasmon resonance (LSPR) effect in the previous research, due to the abundant free charge carrier densities provided by the transition metals [57, 58]. Therefore, to further investigate the enhanced mechanism of metal element doping behavior on the photothermal conversion performance of MXene, the energy band structure and electronic density of states (DOS) of MXene were analyzed using first-principle calculations. In all calculations, the exchange–correlation functional was treated within the Perdew–Burke–Ernzerhof (PBE) method of generalized gradient approximation (GGA), and the OTFG ultrasoft pseudopotential function for electronic structures was adopted to improve accuracy. The cut-off energy was set to 571.4 eV, and the structural relaxations of the MXene supercell were carried out using a 5 × 5 × 1 k-point mesh in the three-dimensional Brillouin zone. Noteworthily, in the previous study, the surface terminal groups, such as specific -O, -F, and -OH of MXene, will have a remarkable effect on the structural, electronic, and electrochemical behavior of MXene, resulting in a large discrepancy between the calculated and experimental properties [59]. Thus, according to the quantified EDS results, the major terminal groups on the MXene surface were the oxygen-containing groups, so the simplified crystal structures of V_2_CO_2_ and (V_1/2_Mo_1/2_)_2_CO_2_ were used instead of V_2_C and (V_1/2_Mo_1/2_)_2_C as the computational modeling to ensure the accuracy (Fig. S10). According to the doping position of the Mo element, the crystal structure of (V_1/2_Mo_1/2_)_2_CO_2_ MXene was set to I and II types, but both were in-plane replacement solid solutions. For the V_2_CO_2_ and (V_1/2_Mo_1/2_)_2_CO_2_ under the number layer of 1, the energy band crossed the Fermi energy level, and no band gap emerged, showing a typical metallic property (Figs. 6c-d and S11). Moreover, the more energy bands and the higher electronic DOS of (V_1/2_Mo_1/2_)_2_CO_2_ MXene than the V_2_CO_2_ can be analyzed near the Fermi energy level, which contributes mainly from the strong localized Mo-4*d* states. Similar to metal materials, the photothermal property of the metallic MXene is closely related to the electronic band structure around EF [45]. Indeed, for the photothermal conversion of MXene materials, the heat energy can be produced concurrently from the Vis-UV and NIR regions, due to the solar absorption properties in the full spectrum (the optical properties curves are shown in Fig. S12). In the Vis-UV regions, the photoexcited hot electrons are governed by interband optical transition, where both electron–electron scattering and electron and/or hole-phonon interaction play important roles in the relaxation of hot electrons [60]. Simultaneously, the photoexcitations in the NIR region are dominated by intraband optical transition, where the relaxation dynamics of hot electrons excited with NIR light are dominated by e-ph interactions, and thermalization is realized by e-ph and h-ph interactions [61]. Distinctly, the higher joint densities of states (JDOS) near EF of (V_1/2_Mo_1/2_)_2_CO_2_ MXene than the V_2_CO_2_ MXene, enabling high populations of hot electrons and holes to be produced through photoexcitation, then leading to a faster relaxation rate and hence to a shorter inelastic lifetime of excited hot electrons, is illustrated in Fig. 6e. Meanwhile, the many energy bands of (V_1/2_Mo_1/2_)_2_CO_2_ MXene also can facilitate the relaxation and heat release of hot carriers [62]. Hence, for (V_1/2_Mo_1/2_)_2_C MXene, the high joint densities of states and multiple energy bands around EF facilitate the process of the photoexcited carrier relaxation and heat release, thereby enhancing the photothermal conversion performance of doped MXene. To verify the effect of the “Mo” element occupancy and number of MXene layers on this mechanism, the crystal structure models of V_2_CO_2_ and (V_1/2_Mo_1/2_)_2_CO_2_ MXene with different occupancy and layers number were constructed, and the corresponding energy band structure and DOS were calculated (Fig. S13). The results show that no matter how to change the occupancy of Mo and V elements, the DOS of “Mo”-doped MXene was always higher than that of pre-doped MXene. Furthermore, the DOS of V_2_CO_2_ MXene and (V_1/2_Mo_1/2_)_2_CO_2_ MXene were always increased multiplied with the increase in the number of layers, and the “Mo”-doped MXene was always higher than those before doping at the same number of layers, even if it is increased to 2 and 3 layers. Therefore, adopting the strategy of transition metal element doping to elevate the electronic DOS near the Fermi level shows a great advantage in boosting the photothermal conversion performance of the MXene.

### Solar Evaporation and Salt-Resistant Performance

**Fig. 7:**
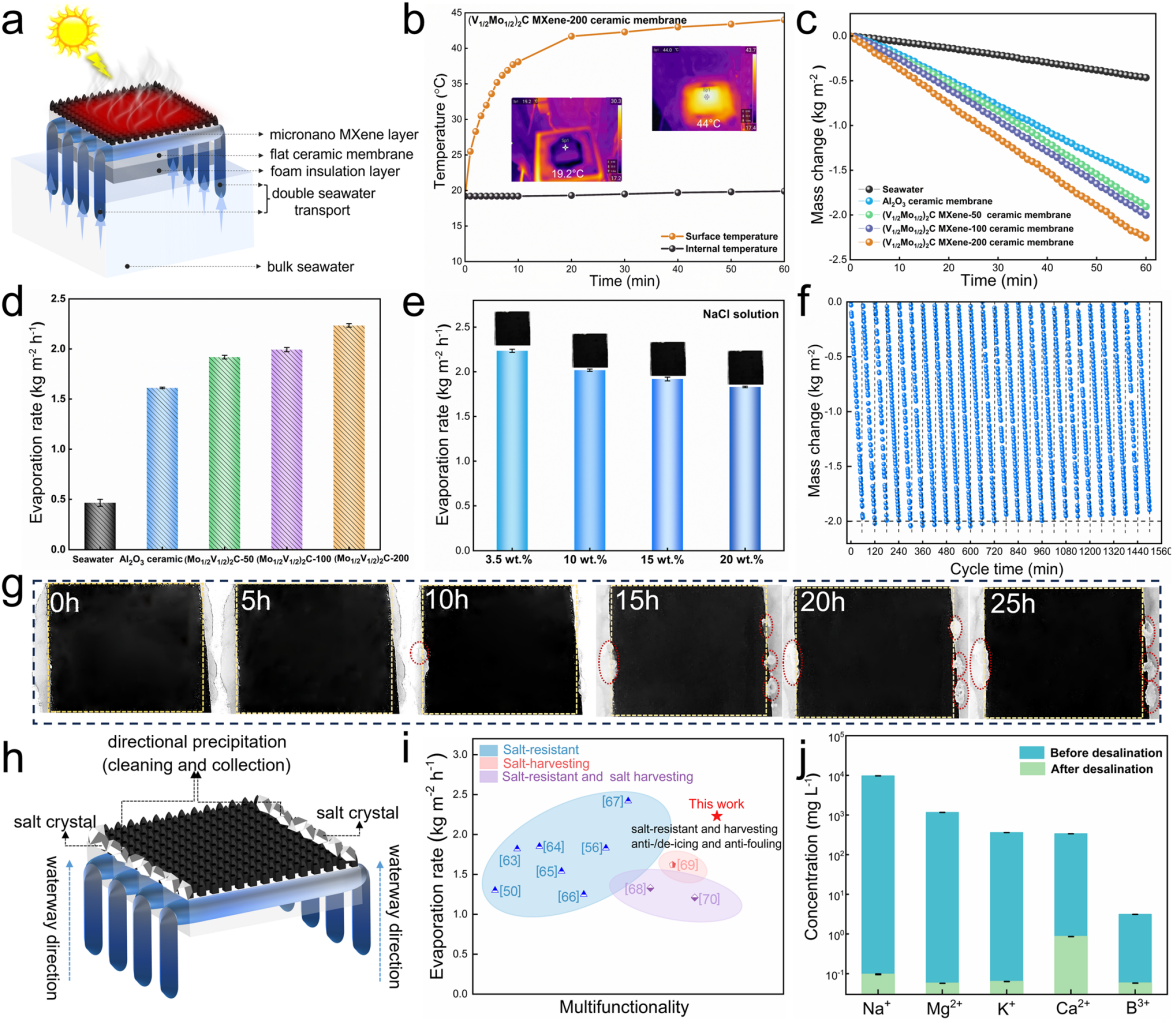
**a** Schematic diagram of the self-assembled evaporator. **b** Surface and internal temperature of (V_1/2_Mo_1/2_)_2_C MXene-200 composite ceramic membrane evaporator. **c** Seawater mass change of all samples under one sun and **d** corresponding evaporation rates. **e** Evaporation rates and surface photographs of (V_1/2_Mo_1/2_)_2_C MXene-200 composite ceramic membrane in different brines. **f** Cycle test of (V_1/2_Mo_1/2_)_2_C MXene-200 composite ceramic membrane under one sun and **g** corresponding surface photographs. **h** Schematic diagram of salt-resistant and directed precipitation. **i** Comparison of evaporation rates in this work with previous reports. **j** Concentrations of typical ions in real seawater and collected freshwater

The solar-driven interfacial evaporation experiments were performed on a self-assembled evaporator (Figs. 7a and S14). Specifically speaking, the composite ceramic membranes were placed on top of the polystyrene (PS) foam, acted as a photothermal conversion layer, and were irradiated by a standard light source of Xe lamp (AM 1.5G). The PS foam acts as the thermal barrier to confine the thermal energy transfer to the bulk seawater, to reduce the heat loss. The cotton cores passed through the parallel array of millimeter channels in the middle of the ceramic membrane, forming a high-throughput two-dimensional water path, with both sides of the membranes simultaneously pumping seawater upward from the bulk seawater below. Furthermore, in the evaporation experiments, the higher environmental temperature will increase the evaporation rate, while too much humidity will weaken the evaporation rate. Therefore, to guarantee the reproducibility of the experimental results, the ambient temperature was adjusted to ≈ 25 °C, and the relative humidity to 60% indoors in this work. After a 60-min irradiation under one sun, the internal water temperature of the evaporator showed only a small increase in 0.6 °C (Fig. 7b), resulting in a low conduction heat loss of 5.4% (Table S2), which is ascribed to the excellent thermal management of air layer inside the ceramic membrane and foam insulation. Furthermore, the seawater mass changes linearly with irradiation time under one sun irradiation, as shown in Fig. 7c. The evaporation rates of samples were calculated basing the mass change (Table S3), and the MXene composite membranes were much higher than that of pure seawater (0.45 kg m^−2^ h^−1^) and original alumina ceramic membrane (1.6 kg m^−2^ h^−1^), and increased significantly with the increase in MXene content. Importantly, the (V_1/2_Mo_1/2_)_2_C MXene-200 composite ceramic membrane achieved the highest evaporation rate of 2.23 kg m^−2^ h^−1^ (Fig. 7d), which was about 4.95 times of pure seawater, proving the excellent solar evaporation performance. The reason can mainly be attributed to the great solar absorption, high photothermal conversion temperature, high-throughput two-dimensional water channels, and excellent thermal management.

The high evaporation performance and structural stability in harsh marine environments are fundamental to the long-lasting working of photothermal materials. Noteworthily, the evaporator will inevitably suffer from the problem of salt buildup contamination by the increased salt concentration, during the constant evaporation process, resulting in the contamination of the photothermal layer and blockage of the vapor escape channels. Thus, designing a photothermal layer with salt-resistant properties and induced salt directional precipitation is of great importance to prevent salt contamination and collect salt for recycling. High salinity and continuous long-cycle evaporation experiments were conducted on the (V_1/2_Mo_1/2_)_2_C MXene-200 composite ceramic membrane to verify the salt-resistant performance and structural stability. As shown in Fig. 7e, NaCl aqueous solutions ranging from 10, 15, and 20 wt% were used to simulate high salinity brine water, and corresponding evaporation rates of 2.01, 1.91, and 1.83 kg m^−2^ h^−1^ were achieved under one sun irradiation, respectively. The high evaporation rates of the evaporator can be still maintained, and no salt was precipitated on the surface of the (V_1/2_Mo_1/2_)_2_C MXene-200 composite membrane. Further, to verify the long-cycle stability of the composite membrane, the 10 wt% brine water was used in a continuous evaporation experiment. Under a continuous 25-h evaporation experiment, a stable evaporation rate of about 2.0 ± 0.2 kg m^−2^ h^−1^ can be achieved (Fig. 7f). The slight fluctuations of data may be influenced by the tiny fluctuation of solar intensity and ambient temperature variation. Remarkable, until continual 10-h irradiation, there was only very little salt crystal that began directionally precipitate at the membrane edge. Meanwhile, with continued solar irradiation, the salt crystallization was gradual accumulation on previous crystals rather than diffusion to the membrane surface, and finally forming a bulk salt at the membrane edge under 25-h irradiation (Fig. 7g). Moreover, the continuous evaporation experiments in real seawater (Huanghai sea, salinity was 3.2 wt%) were further performed on the (V_1/2_Mo_1/2_)_2_C MXene-200 composite membrane. As shown in Fig. S15, no salt was discovered on the surface and edges of the membrane under one sun irradiation for 72 h. When the solar intensity was increased to 800 mW cm^−2^, the salt crystal began to appear at the membrane edge at 5 h, and its volume gradually increased as the evaporation time was extended to 48 h. Noteworthily, even under such high solar intensity, no salt was precipitated on the evaporator surface, further proving the excellent salt resistance and the unique advantage of targeted induction of salt. Undoubtedly, the directed precipitation of the salt bulk can be collected quickly and easily, thereby achieving salt harvesting for recycling, as well as the zero-emission of highly concentrated brine water. Moreover, the evaporation rate of the biomimetic evaporator designed in this work, was superior to many evaporators that focus on the multifunctionality of salt-resistant, salt harvesting, and other functionalities, as shown in Fig. 7i [50, 56, 63–70]. The results confirmed that the photothermal layer can achieve excellent salt-resistant, induced salt directional precipitation properties, and cycle stability of superhydrophobic surface. These advantages can not only avoid evaporation rate degradation due to reduced light absorption, but also enable the recovery and recycling of salt. Conclusively, the excellent properties can be attributed to the construction of biomimetic superhydrophobic surfaces. During the evaporation process, the superhydrophobic composite ceramic membrane can confine the seawater molecules under the photothermal layer, and the salt will not directly contact the top layer of the membrane, thus achieving the salt-resistant performance. Therefore, the seawater at the bottom of the composite membrane was constantly spreading from the center to the surroundings and still was subjected to continuous exchange of upper and lower brine circulations, to keep the salt concentration almost unchanged. In contrast, the seawater at the edge undergoes continuous evaporation to form a higher salinity due to the lack of seawater replenishment, resulting in a salt-oversaturated region, so the precipitates of salt gradually formed and achieved a directional phenomenon, as illustrated in Fig. 7h. Therefore, the aforesaid results are evidence of the significant availability, stability, and excellent endurance of the composite ceramic membrane in harsh marine environments, and have bright prospects for application in actual desalination.

The purity of freshwater is a crucial indication for the seawater desalination technology. In this research, the seawater desalination performance of the natural Huanghai Sea has been estimated utilizing inductively coupled plasma optical emission spectrometry (ICP-OES). As shown in Fig. 7j, the major ion concentrations of Na^+^, Mg^2+^, Ca^2+^, and K^+^ in the natural seawater and freshwater after solar evaporation by (V_1/2_Mo_1/2_)_2_C MXene-200 composite ceramic membrane decreased from 9727.08, 1163.84, 337.31, and 360.95 mg L^−1^ to 0.098, 0.057, 0.867, and 0.063 mg L^−1^, respectively. The results represented a significant reduction in three or four orders of magnitude, achieving a desalination efficiency of 99.99%, which was well below the World Health Organization (WHO) target [71], and China’s drinking water quality standards (GB5749-2006) for drinking water. Remarkably, the concentrations of B^3+^ in the natural seawater, and freshwater after solar evaporation by composite ceramic membrane decreased from 3.13 to 0.057 mg L^−1^, achieving a removal efficiency of 98.17%, which even outperformed the nanofiltration membranes (10–500 ppm) [72]. These results confirmed the outstanding desalination performance of the composite ceramic membrane.

### Anti‐/De‐Icing and Anti-Fouling Performances

**Fig. 8:**
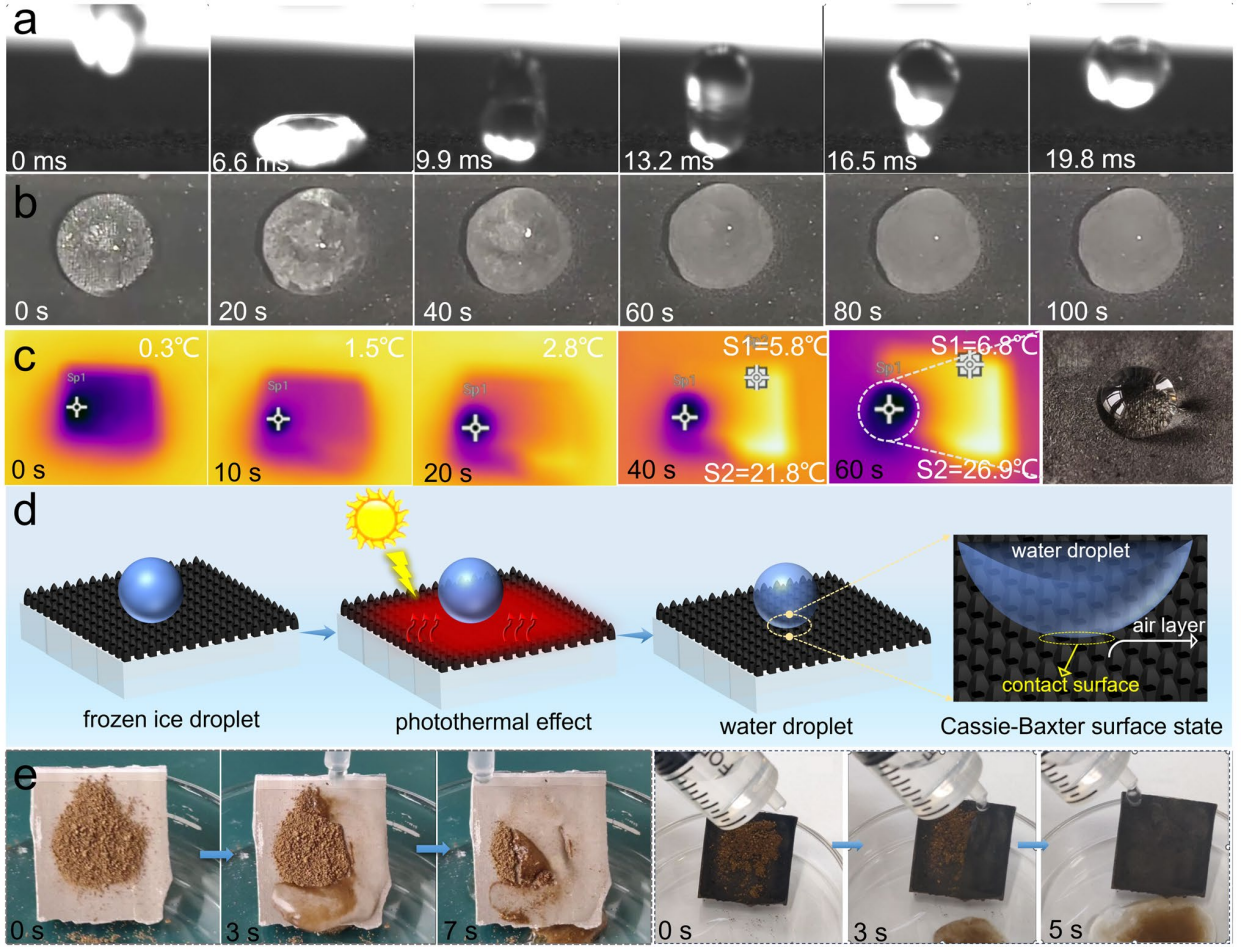
Tests of the multifunctional performance of (V_1/2_Mo_1/2_)_2_C MXene-200 composite ceramic membrane. **a** Typical snapshots of water droplets impacting composite ceramic membrane surface. **b** Typical images of a water droplet (≈10 μL) freezing on the superhydrophobic composite ceramic membrane surface at -40 °C. **c** Surface temperature evolution of the composite ceramic membrane under Xe lamp irradiation over time. **d** Schematic diagram of a photothermal de-icing mechanism for the composite ceramic membrane. **e** Typical images of composite ceramic membrane contaminated by sands before and after droplets washing over time

Practical application conditions are always complex and variable, and maintaining excellent stability, anti-/de-icing, anti-fouling, and antimicrobial performances in extreme environments is particularly important. To test the stability of the superhydrophobic surface, a droplet impacting experiment was performed on the (V_1/2_Mo_1/2_)_2_C MXene-200 composite ceramic membrane. At a height of 10 cm from the surface, the water droplets were allowed to fall freely to impact the surface, and a typical droplet rebound phenomenon was observed (Fig. 8a), which indicated the robust superhydrophobicity of the composite ceramic membrane surface. To evaluate the anti‐/de‐icing performance of composite ceramic membrane in real environments, the original ceramic membrane and (V_1/2_Mo_1/2_)_2_C MXene-200 composite ceramic membranes were set at a -40 °C extremely low-temperature environment for 5 h, and then the delayed icing behavior on the surface was investigated. The water droplet was frozen for a very short time of 25 s on the hydrophilic original ceramic membrane surface and showed a semi-spreading state (Figs. 8b and S16). In contrast, on the superhydrophobic surface, it took 100 s for water droplets from contact with each other to freeze into an ice bead, and a spherical ice bead remained after totally freezing, showing a delayed freezing time on the superhydrophobic surface was 4 times longer than that on the hydrophilic surface. The reason can be attributed to the different contact areas of water and surface on the superhydrophobic and hydrophilic surface states. The hydrophilic surface is high that leads to the rapid spreading of water droplets on the surface, augmenting the contact area, which in turn leads to an increase in the nucleation density and nucleation sites of ice crystals, facilitating the rapidly agglomerates and growth of ice nuclei, resulting in the fast formation of an ice surface. In contrast, on the superhydrophobic surface, the existing gaps in the micro-nanostructures can provide additional air layers to reduce the contact area of the water droplet with the surface, achieving delayed icing. Moreover, the photothermal de-icing property of the composite membrane surface was further investigated based on the excellent photothermal conversion performance of MXene. As shown in Fig. 8c, the composite ceramic membrane surface was heated up rapidly with irradiation time under an Xe lamp, and the ice bead can be heated from 0 to 6.8 °C and completely melted into water droplets in just 60 s. Importantly, the melted water droplet remained on the membrane surface and did not penetrate, exhibiting a Cassie–Baxter surface state, as illustrated in Fig. 8d. The results demonstrated the excellent passive photothermal de-icing property of the composite membrane. The above experiments not only proved that the effective anti-icing and photothermal de-icing performance can be achieved by the composite membrane, but also proved that the great stability of superhydrophobicity can be maintained at both ultra-low and high temperatures, which provides more possibilities for its practical application in intricate service environments.

To verify the anti-fouling property, the sand pollutions were simulated on the hydrophilic alumina ceramic membrane and superhydrophobic (V_1/2_Mo_1/2_)_2_C MXene-200 composite ceramic membrane surface. As shown in Fig. 8e, the sands were pre-spread on the surface of the tilted composite ceramic membrane, and the deionized water was subsequently dripped from above it. On the hydrophilic membrane surface, when dry sands come into contact with water, it becomes moist sediment attached to the hydrophilic surface, with only a small amount of sediment being washed into the glass container below. In contrast, the sand on the superhydrophobic surface can be easily dislodged by the water flow within a short time, demonstrating excellent resistance to sediment contamination. The excellent anti-fouling and self-cleaning properties are due to the small rolling angle (3° ± 0.2°) of the superhydrophobic surface. More importantly, the antimicrobial property of the solar desalination technology-based superhydrophobic surface has attracted increasing attention. To evaluate the antimicrobial performance of superhydrophobic (V_1/2_Mo_1/2_)_2_C MXene-200 composite ceramic membrane, the real seawater and collected freshwater after desalination by membrane were used to perform bacterial testing. After wetting the total bacterial agar medium with different water samples, and incubating the bacteria for the same time and temperature, the obvious differences were found that the red bacterial spots appeared on the surface of the medium wetted with seawater, whereas no bacterial spots appeared on the medium wetted with collected freshwater, proving the excellent antibacterial property of the composite ceramic membrane synergistic solar desalination technology (Fig. S17). The antimicrobial mechanisms may be attributed to three aspects: firstly, the superhydrophobic composite membrane surface reduces the contact area between the bacteria and the material surface, preventing the adhesion of the bacteria; secondly, the rough micro-nanostructures on the membrane surface, and inherently sharp 2D layer structures edges of MXene can insert into pathogenic cells, resulting in bacterial death [73]; thirdly, the high-temperature vapor during the photothermal conversion process facilitated the ablation of bacterial structures, resulting in bacterial inactivity. Therefore, the synergistic existence of the three reasons confers multiple antimicrobial effects in terms of anti-adhesion and bactericidal bacteria for superhydrophobic composite ceramic membranes.

## Conclusions

In summary, we designed and fabricated a novel (V_1/2_Mo_1/2_)_2_CT_x_ MXene composite membrane, with biomimetic micro-nanostructures and photothermal superhydrophobic surface by laser etching technology. Noteworthily, the doping of transition metal element at the “M”-point position significantly enhances the photothermal conversion performance via elevated joint densities of states, enabling high populations of photoexcited carrier relaxation and heat release. Furthermore, the design of the micro-nanostructure surface not only effectively increases the “light trap” effect via extending the refraction path of light, but also significantly contributes to the bonding strength between MXene and ceramic substrate via the “mechanical interlocking” effect. Moreover, the (V_1/2_Mo_1/2_)_2_CT_x_ MXene-200 composite membrane can achieve a high evaporation rate of 2.23 kg m^−2^ h^−1^ under one sun irradiation, which benefits from the excellent photothermal conversion performance, high-throughput water transportation of two-dimensional arrayed waterways, and effective thermal management. In addition, the superhydrophobic surface can induce the directional precipitation of salt crystals at the membrane edge, thus enabling salt harvesting for recycling, and zero-emission of highly concentrated brine water as well. More importantly, the composite membrane was endowed with outstanding versatility including anti‐/de‐icing, anti-fouling, and antibacterial properties, attributed to the ingenious construction of a superhydrophobic surface with micro-nanostructure. Therefore, the durability, stability, and multifunctional of composite membranes can contribute to the longevity and reliability of solar desalination under tanglesome marine environments, which is a promising and highly efficient candidate for the cost-effective production and practical application.

## Supplementary Information

Below is the link to the electronic supplementary material.

Supplementary file1 (DOCX 10146 kb)

Supplementary file2 (MP4 23685 kb)

Supplementary file3 (MP4 26335 kb)

Supplementary file4 (MP4 22376 kb)
