# Supplementary material for: Biomimetic Micro-Nanostructured Evaporator with Dual-Transition-Metal MXene for Efficient Solar Steam Generation and Multifunctional Salt Harvesting
Source: Nanomicro Lett. 2025 Jan 6;17:102. doi: 10.1007/s40820-024-01612-0 (PMC11704123; doi:10.1007/s40820-024-01612-0)
Supplement: Supplementary file 1 — Supplementary file1 (DOCX 10146 kb) [file 40820_2024_1612_MOESM1_ESM.docx]

Supporting Information for

**Biomimetic Micro-Nanostructured Evaporator with Dual-Transition-Metal MXene for Efficient Solar Steam Generation and Multifunctional Salt Harvesting**

Ruiqi Xu^1^, Hongzhi Cui^1,^ *, Na Wei^1,2^, Yang Yu^1^, Lin Dai^1^, Xiaohua Chen^1^

^1^ College of Materials Science and Engineering, Ocean University of China, Qingdao 266100, People’s Republic of China

^2^ College of Materials Science and Engineering, Shandong University of Science and Technology, Qingdao 266590, People’s Republic of China

*Corresponding author. E-mail: [cuihongzhi@ouc.edu.cn](mailto:cuihongzhi@ouc.edu.cn) (Hongzhi Cui)

**S1 Experimental**

**S1.1** **Solar evaporation and desalination experiments**

The solar-driven interfacial evaporation experiments were performed on a self-assembled evaporator and self-constructed evaporation system. Specifically speaking, the evaporator consists of an acrylic box, a composite ceramic membrane, cotton wicks, polystyrene (PS) foam, and bulk seawater from top to bottom. Among them, the acrylic box was the seawater container, the composite membranes as a photothermal conversion layer (the size of the membrane was 30*30 mm), and the cotton wicks passed through a parallel array millimeter channel in the middle of the ceramic membrane, and naturally hang down into the water from both ends, result in the 2D array channel to pump seawater from bottom to up. The PS foam acts as the thermal barrier to confine the thermal energy transfer to the bulk seawater, to reduce the heat loss.

In the evaporation system, a standard light source (HM-Xe 500 W) was used to simulate solar irradiation in the full spectrum, and the solar intensity was detected via an optical power meter (CEL-NP2000). The surface temperature of photothermal conversion membranes was measured by an infrared camera (Fotric, 320C, China), and the internal temperature of the evaporator was tested by a thermocouple. The mass change during the evaporation process was recorded by an electronic analytical scale (FA2004, 0.1 mg accuracy) connected to a computer.

Seawater in this work came from the Huanghai Sea. The ion concentrations of real seawater and collected freshwater were tested by an analytical plasma emission spectrometer (ICP-OES/MS, SPECTRO).

**S1.2** **Antimicrobial test**

The agar bacterial media used in this work was obtained from PYXIS Ltd., the individual media strains include Escherichia coli, Legionella, Staphylococcus aureus, Bacillus cereus, Bacillus licheniformis, and Salmonella. During the experiment, the culture medium of model number Dipslide AB-T was submerged in real seawater from the Huanghai Sea and collected freshwater after desalination, respectively. Subsequently, the medium was taken out and incubated in a constant-temperature oven at 37 °C for 48 h, to observe the growth of colonies on its surface.

**S2 XRD diffraction pattern**


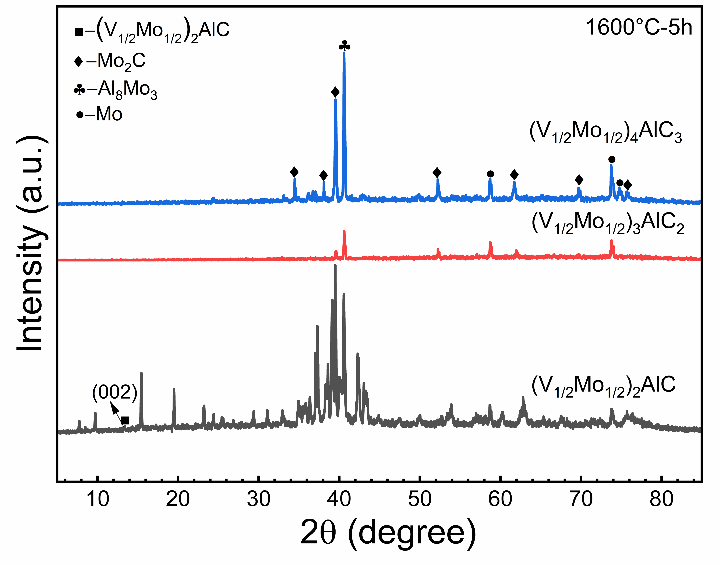


**Fig. S1** XRD diffraction pattern of (V_1/2_Mo_1/2_)_2_AlC, (V_1/2_Mo_1/2_)_3_AlC_2_, and (V_1/2_Mo_1/2_)_4_AlC_3_ MAX phase under sintering temperature at 1600 °C for 5h

**S3 XPS spectrums**


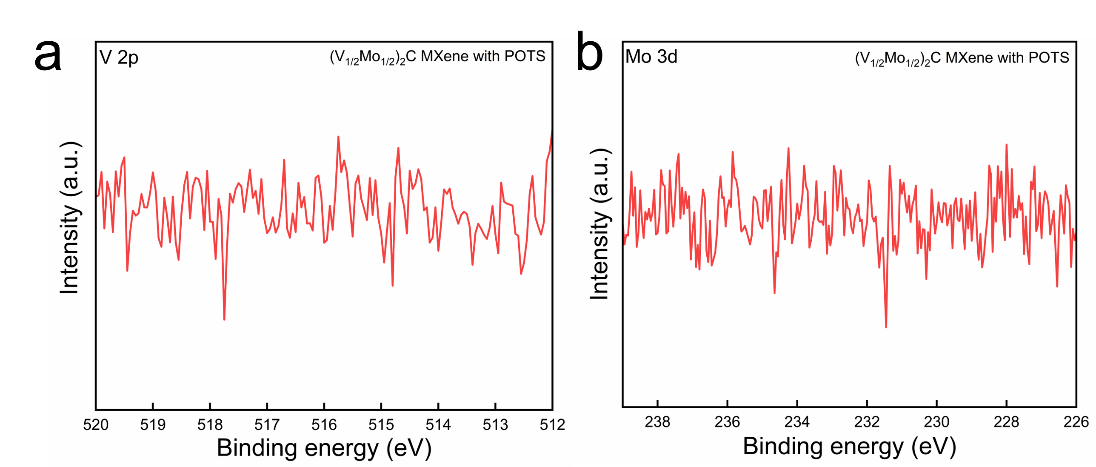


**Fig. S2** XPS spectrum of (V_1/2_Mo_1/2_)_2_CT_x_ MXene after POTS modification: **a** V 2p, **b** Mo 3d

**S4 SEM images**


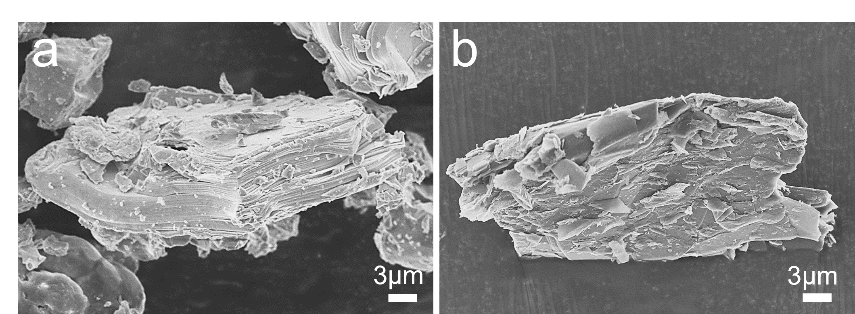


**Fig. S3** SEM images of MAX phase under sintering temperature at 1600 °C for 4h of **a** (V_1/2_Mo_1/2_)_3_AlC_2_, **b** (V_1/2_Mo_1/2_)_4_AlC_3_


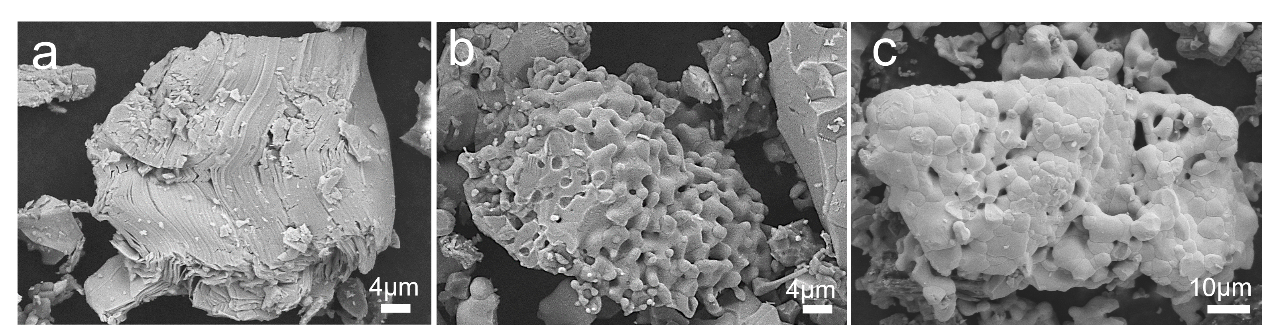


**Fig. S4** **a-c** SEM images of (V_1/2_Mo_1/2_)_2_AlC, (V_1/2_Mo_1/2_)_3_AlC_2_, and (V_1/2_Mo_1/2_)_4_AlC_3_ MAX phase under sintering temperature at 1600 °C for 5h


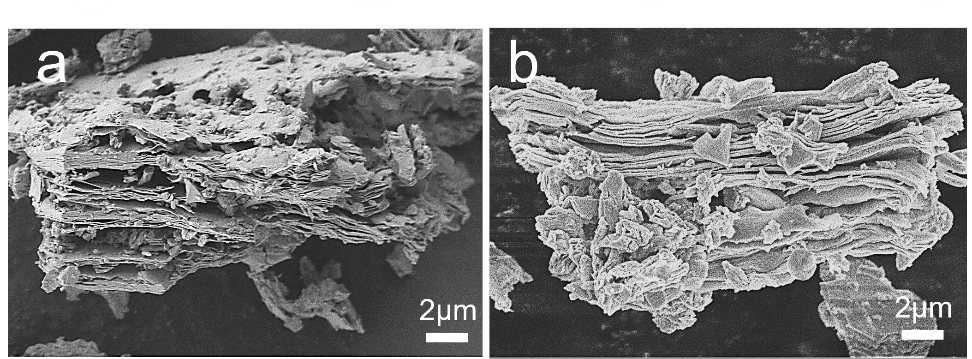


**Fig. S5** SEM images of MXene at an etching temperature of 100 °C: **a** (V_1/2_Mo_1/2_)_2_CT_x_ MXene-4h, **b** (V_1/2_Mo_1/2_)_2_CT_x_ MXene-5h

**S5 Calculation of crystal plane orientation**

The calibration of crystal plane orientation in this work is divided into two main steps. Firstly, the Digital Micrograph software was used to measure the spacing of multiple lattice fringes in the HRTEM results, and the average was interplanar spacing to minimize errors. Further, the measured interplanar spacing by HRTEM was compared to the XRD diffraction patterns of known phases, to determine the indices of the crystallographic plane via the PDF standard card. In this work, the measured interplanar spacing of (V_1/2_Mo_1/2_)_2_C MXene was 1.211 nm via the HRTEM results, and the experimental result from XRD diffraction patterns was 1.207 nm, corresponding to the typical (002) crystal plane of MXene. The tiny differences are measurement errors. Then, the Fourier transform and inverse Fourier transform processes were performed on localized regions to reduce the noise of the image, thereby achieving clearer structural information and facilitating a more accurate analysis of the crystal structure. Meanwhile, the interplanar distance of localized enlarged regions of (V_1/2_Mo_1/2_)_2_C MXene was measured to be 0.226 nm, corresponding to the (100) crystal plane. Secondly, to further verify the accuracy of the experimental results and simulation calculations, the (002) crystal plane of the constructed (V_1/2_Mo_1/2_)_2_C MXene crystal structure model was intercepted to make an alignment with the atomic plane from HRTEM results. The results showed that both the crystal planes displayed a high degree of consistency, confirming the accuracy of crystal structure models and basal plane hexagonal symmetry structure of the multilayer (V_1/2_Mo_1/2_)_2_C MXene.

**S6 Atomic percentage of elements in MXene**

**Table S1** Atomic percentage of elements in MXene

| **Elements** | **Atomic Fraction (%)** | **Atomic Error (%)** |
| --- | --- | --- |
| V | 16.72 | 3.25 |
| Mo | 14.30 | 2.29 |
| C | 47.35 | 5.29 |
| O | 13.19 | 3.02 |
| F | 8.44 | 1.94 |

**S7 Performance of ceramic membranes before PDMS modification**

**
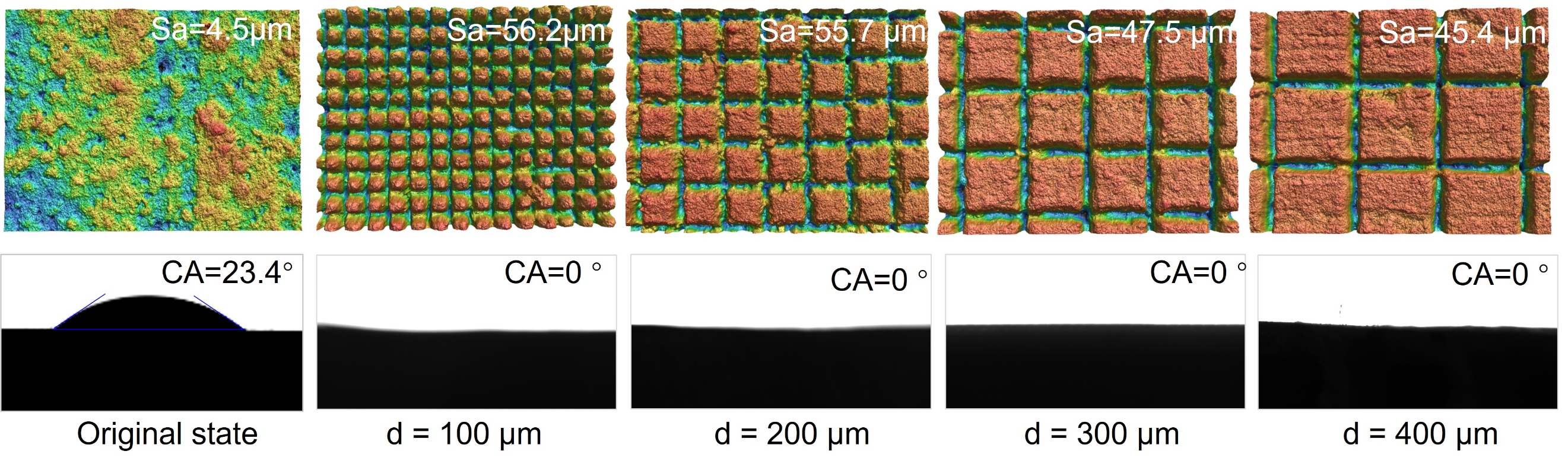
**

**Fig. S6** The surface three-dimensional morphology, surface roughness, and contact angle pictures of the original alumina ceramic membrane and micron-scale structure ceramic membranes before PDMS modification

**S8** **SEM images and stability experiments of composite ceramic membranes**


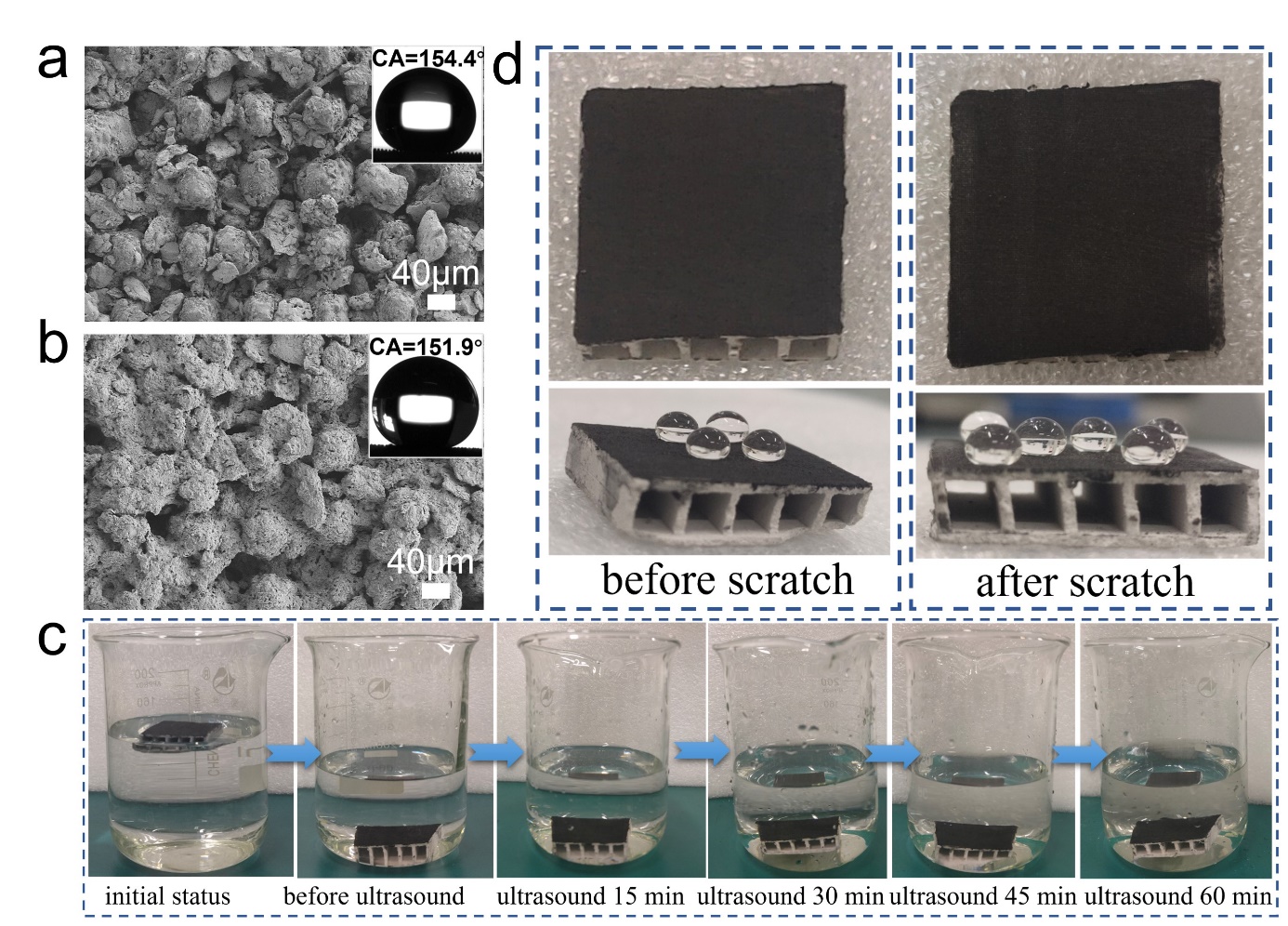


**Fig. S7** The SEM images and surface contact angle pictures of MXene/alumina composite ceramic membranes, with 100 μm groove distance and different MXene content of **a** (V_1/2_Mo_1/2_)_2_C MXene-50, **b** (V_1/2_Mo_1/2_)_2_C MXene-100. **c** Continuous sonication test of (V_1/2_Mo_1/2_)_2_C MXene-200 membrane. **d** The sandpaper scratch test of (V_1/2_Mo_1/2_)_2_C MXene-200 membrane

**S9** **Surface roughness and optical properties of samples**


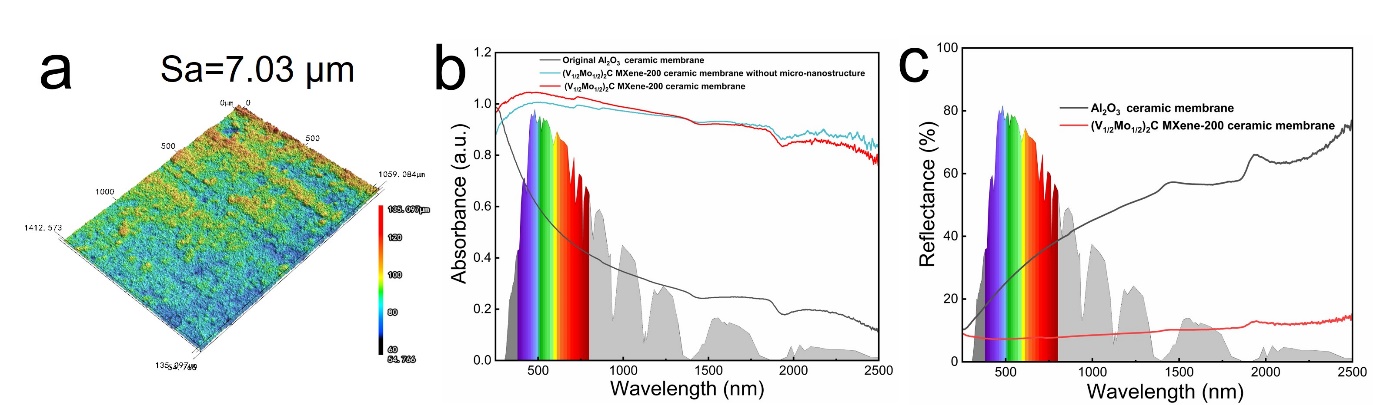


**Fig. S8 a** The surface three-dimensional morphology and surface roughness of alumina flat ceramic membrane without groove and MXene. **b-c** The UV-Vis-NIR spectrums of all composite ceramic membranes: **b** absorbance, **c** reflectance

**S10 IR images of V_2_C MXene**


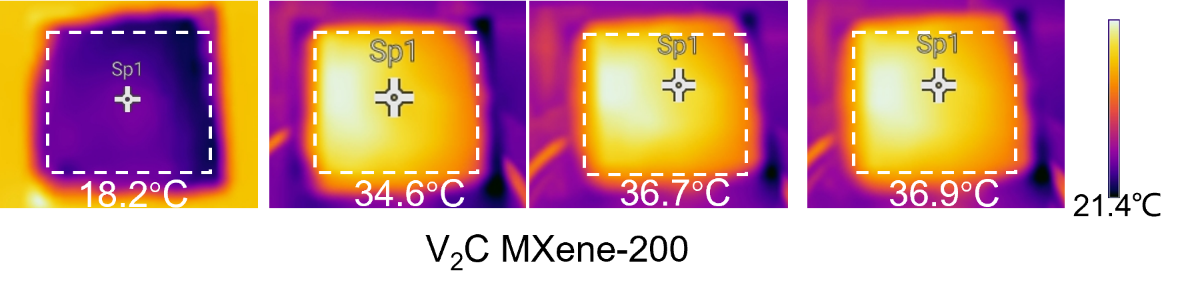


**Fig. S9** The IR images of V_2_C MXene-200 under one-sun irradiation in 1 h

**S11 Crystal structure models**


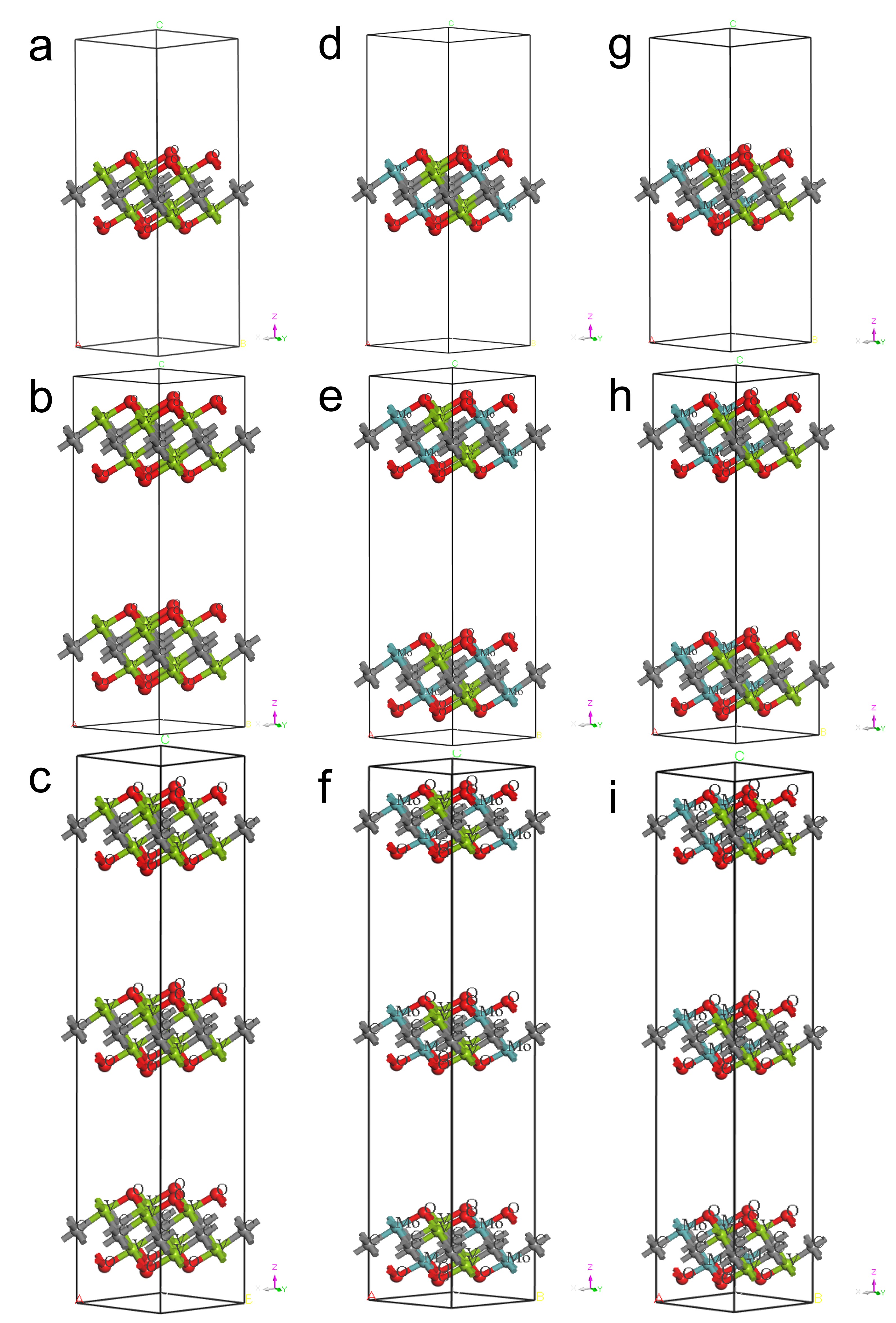


**Fig. S10** The crystal structure models of V_2_CO_2_ MXene of different layers of **a-c** 1, 2, and 3 layers. The crystal structure models of (V_1/2_Mo_1/2_)_2_CO_2_ of different element occupancy and number of layers of **d-f** I type of 1, 2, and 3 layers; **g-i** II type of 1, 2, and 3 layers

**S12 DFT calculation results of (V_1/2_Mo_1/2_)_2_CO_2_ MXene**

**

**

**Fig. S11** The energy band structure and electronic density of state (DOS) results of (V_1/2_Mo_1/2_)_2_CO_2_ MXene under 1 layer (II type)

**S13 Optical properties of MXene**


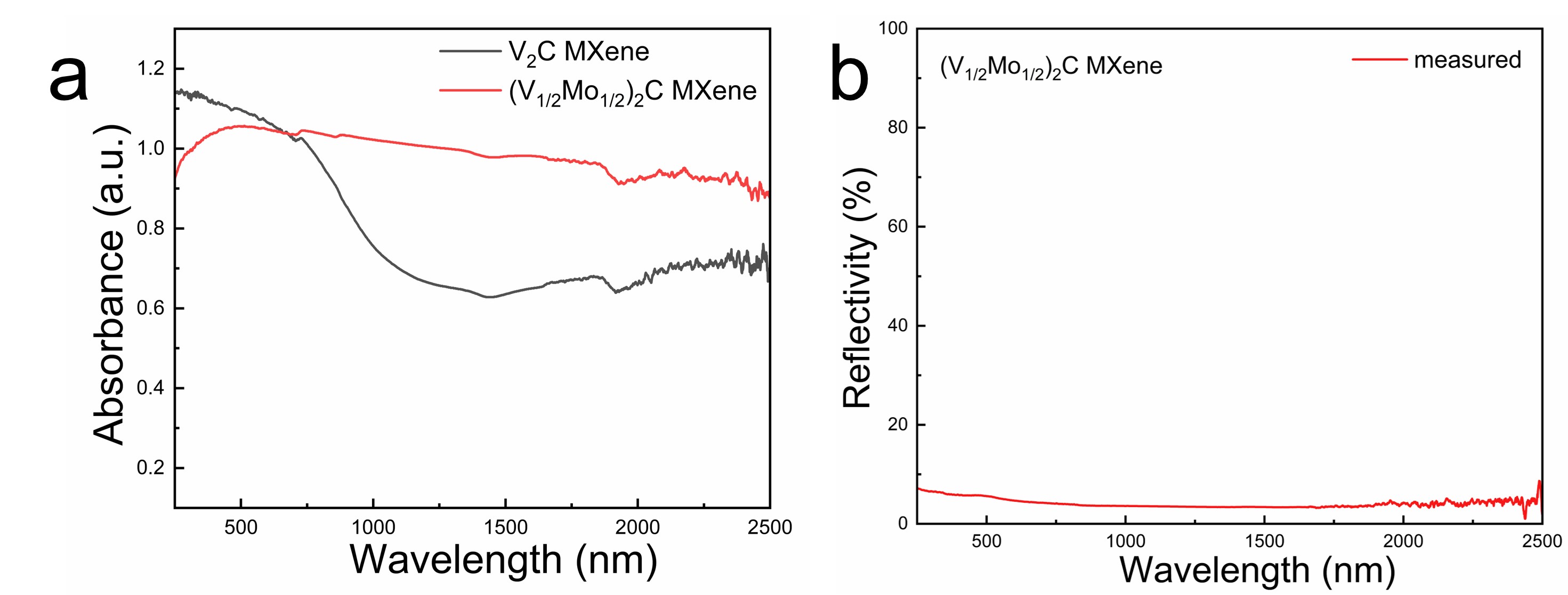


**Fig. S12 a** The solar absorbance of V_2_C MXene and (V_1/2_Mo_1/2_)_2_C MXene powders. **b** The reflectivity of (V_1/2_Mo_1/2_)_2_C MXene powders

**S14 DFT calculation results of V_2_CO_2_ and (V_1/2_Mo_1/2_)_2_CO_2_ MXene**


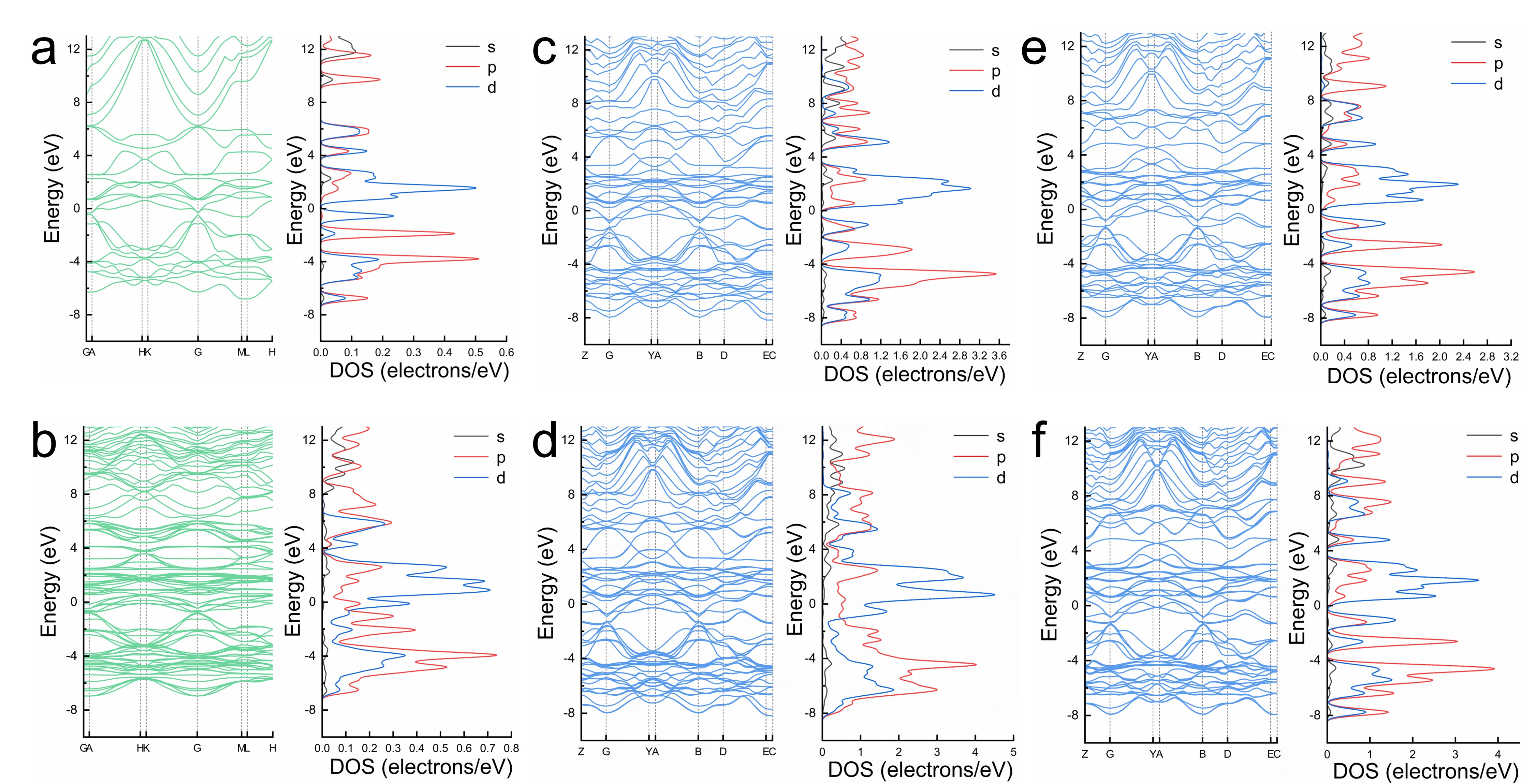


**Fig. S13** The energy band structure and electronic density of state (DOS) results of **a-b** V_2_CO_2_ MXene of 2 and 3 layers**; c-d** (V_1/2_Mo_1/2_)_2_CO_2_ MXene of 2 and 3 layers (I type), and **e-f** (V_1/2_Mo_1/2_)_2_CO_2_ MXene of 2 and 3 layers (II type)

**S15** **Evaporator and evaporation system**


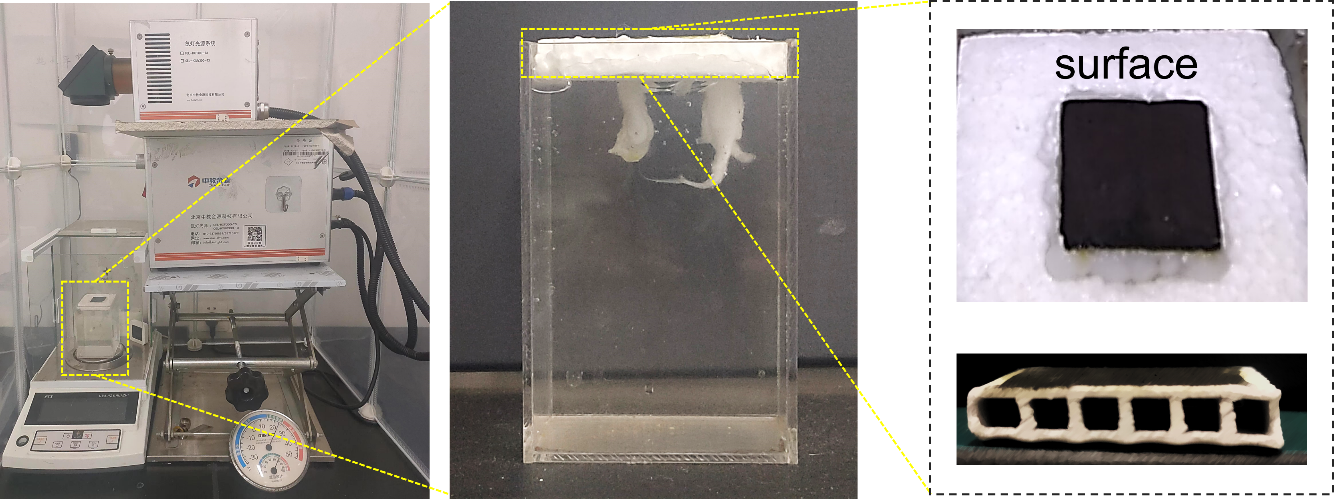


**Fig. S14** Photographs of the self-assembled evaporator and evaporation system

**S16 Calculation of the heat loss [S1-S4]**

a) Radiation loss:

The radiation loss is calculated by the Stefan-Boltzmann equation.

$\emptyset=\varepsilon A\sigma({T_{1}}^{4}{{-T}_{2}}^{4})$ (S1)

*Φ* is heat flux (W), *ε* is the emissivity (0.9), *A* is the surface area of photothermal material (9 cm^2^), *σ* is the Stefan-Boltzmann constant (5.67×10^-8^ W m^-2^ K^-4^), *T_1_* is the surface temperature of the (V_1/2_Mo_1/2_)_2_C MXene-200 composite membrane under one-sun illumination after 1h (317.155 K), and *T_2_* is the ambient temperature around the evaporator (310.15K). Therefore, based on the Equation, we can calculate that the radiation heat loss is ∼4.4 %.

b) Convection loss:

Convective heat loss is defined by Newton's law of cooling.

$Q=hA\Delta T$ (S2)

*Q* is the heat energy, *h* is the convection heat transfer coefficient (5 W m^-2^ K^-1^), and *A* represents the surface area of photothermal material (9 cm^2^). *ΔT* (2.5 K) is the difference value between the surface temperature and steam temperature of the (V_1/2_Mo_1/2_)_2_C MXene-200 composite membrane. The convection heat loss is ∼1.25%.

c) Conduction loss:

The heat loss of convection was calculated according to the following Equation.

$Q=Cm\Delta T$ (S3)

*Q* is the heat energy, *C* is the specific heat capacity of pure water (4.2 kJ ^°^C^-1^ kg^-1^), and *m* is the weight of bulk seawater (70 g). *ΔT* (0.6 ^°^C) is the temperature difference value of bulk seawater between before and after solar illumination. Therefore, we can calculate that the conduction heat loss is ~5.4%.

**Table S2** The calculation results of heat loss

| Heat energy | Energy (J) | Input energy (J) | Heat loss (%) |
| --- | --- | --- | --- |
| Radiation | 142.86 | 3240 | 4.4 |
| Convection | 40.5 | 3240 | 1.25 |
| Conduction | 176.4 | 3240 | 5.4 |

**S17 Calculation of solar absorption and evaporation rate** **[S1-S4]**

a) Absorption:

*A = 100 – R-T* (S4)

where *A* (%) is the absorption, *R* (%) is the reflection, and *T* (%) is the transmission**.** The ceramic membranes in this work are thicker and it is difficult for the light to pass through, so the T = 0.

b) Evaporation rate:

The formula for calculating the seawater evaporation rate was

$m=\Delta m\div A$ (S5)

Where *A* is the test area of the membrane.

c) Photothermal conversion efficiency:

The formula for calculating solar steam conversion efficiency is as follows：

$\eta=m^{'}\times\left( L_{V}+Q \right)\div P_{in}$(S6)

where *m* (kg m^-2^ h^-1^) is the evaporation rate, *∆m* (kg) is the mass loss measured for 1h, *A* is the test area of the photothermal material (m^2^), *η* (%) is energy conversion efficiency, and *m'* is the evaporation rate under light conditions subtracted from that under dark conditions. *Lv* is the latent heat of the phase transformation of water, and *Q* is the sensible heat of the phase transition per unit mass of water. *Q = c × (T_2_ -T_1_)*; the unit of *Q* is (kJ kg^-1^), and *c* is the specific heat capacity of water. *T_1_* is the initial temperature of the water, and *T_2_* is the evaporation temperature. The *P_in_* is the total heat input toward the photothermal material, *P_in_=P×A,* A is the surface area of the photothermal material (9 cm^2^), and P is the optical power density (100 mW cm^-2^).

**Table S3** The evaporation rate under one sun of all samples

| **Samples** | **Seawater** | **Original ceramic membrane** | **(V_1/2_Mo_1/2_)_2_C MXene-50 composite membrane** | **(V_1/2_Mo_1/2_)_2_C MXene-100 composite membrane** | **(V_1/2_Mo_1/2_)_2_C MXene-200 composite membrane** |
| --- | --- | --- | --- | --- | --- |
| **Evaporation**  **Rate**  **(kg m^-2^ h^-1^)** | 0.45 | 1.6 | 1.92 | 1.99 | 2.23 |

**S18 Continuous evaporation test in real seawater**


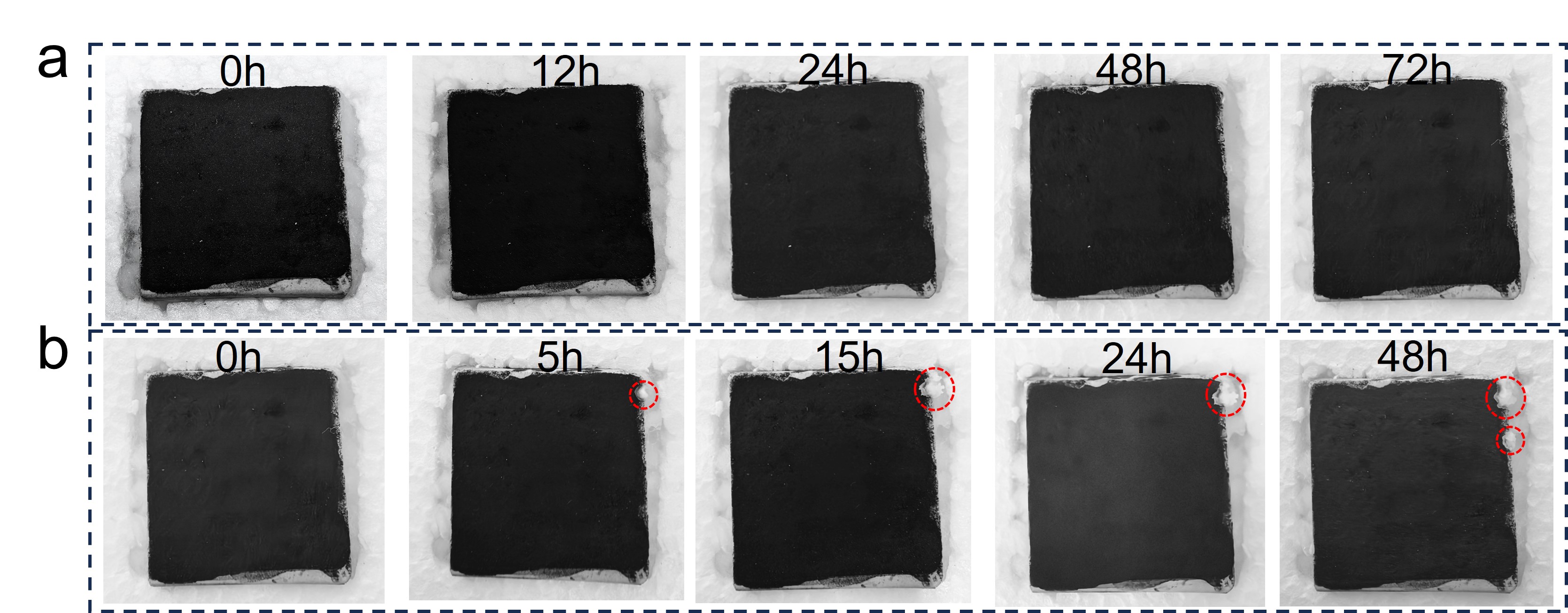


**Fig. S15** The continuous evaporation test of the (V_1/2_Mo_1/2_)_2_C MXene-200 composite ceramic evaporator in real seawater. **a-b** Photographs of the membrane surface under different solar intensity of **a** 100 mW cm^-2^ and **b** 800 mW cm^-2^

**S19** **Icing behavior of water droplets**


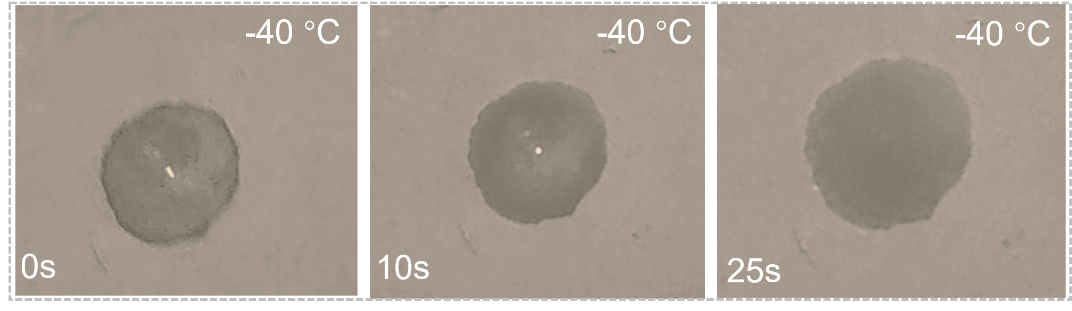


**Fig. S16** Typical images of a water droplet (≈10 μL) freezing on the hydrophilic surface of the ceramic membrane without treatment at -40 °C

**S20** **Antibacterial experiment**


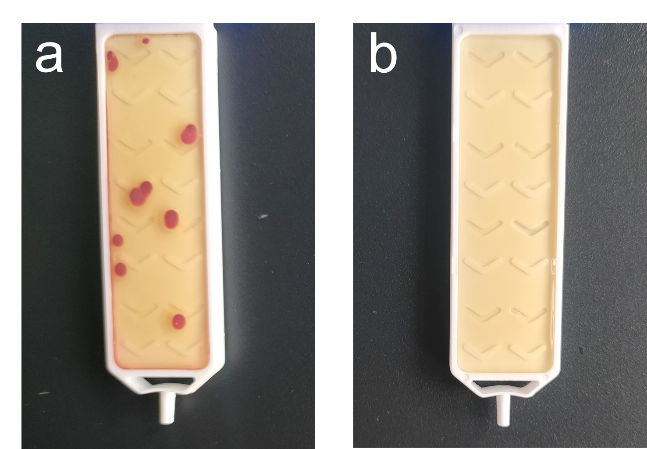


**Fig. S17** Bacterial culture pictures of **a** Seawater and **b** Collected freshwater

**Supplementary References**

1. X. Li, R. Lin, G. Ni, N. Xu, X. Hu et al., Three-dimensional artificial transpiration for efficient solar waste-water treatment. Natl. Sci. Rev. **5**(1), 70 (2018). <https://doi.org/10.1093/nsr/nwx051>
2. S. Liu, C. Huang, X. Luo, C. Guo, Performance optimization of bi-layer solar steam generation system through tuning porosity of bottom layer. Appl. Energy **239**, 504-513 (2019). <https://doi.org/10.1016/j.apenergy.2019.01.254>
3. P.-F. Liu, L. Miao, Z. Deng, J. Zhou, Y. Gu et al., Flame-treated and fast-assembled foam system for direct solar steam generation and non-plugging high salinity desalination with self-cleaning effect. Appl. Energy **241**, 652-659 (2019). <https://doi.org/10.1016/j.apenergy.2019.02.030>
4. N. Li, L. Qiao, J. He, S. Wang, L. Yu et al., Solar-driven interfacial evaporation and self-powered water wave detection based on an all-cellulose monolithic design. Adv. Funct. Mater. **31**(7), 2008681 (2020). <https://doi.org/10.1002/adfm.202008681>
